# Sexually dimorphic gene expression in the lateral eyes of *Euphilomedes carcharodonta* (Ostracoda, Pancrustacea)

**DOI:** 10.1186/s13227-015-0026-2

**Published:** 2015-11-10

**Authors:** Andrea Sajuthi, Brenna Carrillo-Zazueta, Briana Hu, Anita Wang, Logan Brodnansky, John Mayberry, Ajna S. Rivera

**Affiliations:** Department of Biological Sciences, University of the Pacific, Stockton, CA USA; Stritch School of Medicine, Loyola University, Chicago, IL USA; Dugoni School of Dentistry, University of the Pacific, San Francisco, CA USA; Thomas J. Long School of Pharmacy and Health Sciences, University of the Pacific, Stockton, CA USA

**Keywords:** Sexual dimorphism, Evolution, Development, Eye, Expression, Pancrustacean, Ostracod

## Abstract

**Background:**

The evolution and development of sexual dimorphism illuminates a central question in biology: How do similar genomes produce different phenotypes? In an XX/XO system especially the state of a sexually dimorphic trait is determined by differences in gene expression, as there are no additional genetic loci in either sex. Here, we examine the XX/XO ostracod crustacean species *Euphilomedes carcharodonta*. This species exhibits radical sexual dimorphism of their lateral eyes, females have only a tiny simple lateral eye while males have elaborate ommatidial eyes.

**Results:**

We find that males express three of nine eye-development gene homologs at significantly higher levels during juvenile eye development, compared to females. We also find that most eye-development genes examined are pleiotropic, with high expression levels during embryonic development as well as during juvenile eye development. Later, in adults, we find that phototransduction genes are expressed at higher levels in males than in females, as we might expect when comparing ommatidial to simple eyes.

**Conclusions:**

We show here that expression changes of a handful of developmental genes may underlie the radical difference in a dimorphic character. This work gives an important point of comparison for studying eye evolution and development in the Pancrustacea.

**Electronic supplementary material:**

The online version of this article (doi:10.1186/s13227-015-0026-2) contains supplementary material, which is available to authorized users.

## Background

In recent years, the genetics underlying sex determination have been under close scrutiny. Deep homology has been found at the heart of the diverse regulatory systems underlying sex determination in disparate animals [rev. in 1]. However, the output of these gene regulatory networks, the genetic underlying specific sexually dimorphic traits, has been more elusive. Studying sexual dimorphism at the genetic level allows us to ask two intriguing questions. First, in determining a dimorphic character, how does a single set of genes found within the same species produce multiple phenotypes among individuals of different sexes? Recent studies focused on the global expression differences in males and females have found hundreds to thousands of genes expressed in a sexually dimorphic fashion [2–4]. On a different scale, other studies have uncovered the expression differences in a single gene underlying fate choice of a bipotential tissue, finding that dimorphic expression is key to fate determination [5–8].

The second question is an evolutionary one: How does dimorphism evolve at the genetic level, and how are two phenotypes maintained in a single species? The evolution of dimorphism has been studied at the genetic level in other organisms and has led to gains in understanding both the genetics of trait evolution as well as the genetic basis of convergence. Work on the *Drosophila* pigmentation gene *bric*-*a*-*brac* (*bab*) has revealed an evolutionary scenario for changes in axial patterning. In this case, ancestral monomorphic expression evolved into a dimorphic pattern via complex changes in cis-regulatory elements regulated by the axial patterning gene *abd*-*B* and the sex-determination gene *doublesex* (*dsx*) [6]. Another group of insects, butterflies, also have species with sexually dimorphic pigmentation patterning. In the case of *Heliconius*, the gene expression difference in *optix* that underlies the dimorphism in *H. doris* also underlies much morphological diversity among the genus [9]. The genetic link between sexual dimorphism and macroevolution is not limited to insects, recent studies on cichlid fish have shown that sexually dimorphic craniofacial morphology is similar to interspecies variation and may have a similar developmental basis [10, 11].

Pancrustacean eyes are a particularly useful case for studying the evolution and development of eyes at the genetic level. Not only do they exhibit the largest number of optical types of all animal groups [12] but they also have a large number of genetic tools available and a model genetic organism to compare findings to, *Drosophila*. Moreover, unlike other examples of sexual dimorphism informing macroevolution, compound eyes in Pancrustacea are complex structures; understanding the genetics underlying eye diversity can deepen our understanding of eye evolution in general. Currently, the evolution of image-forming eyes in arthropods is an unresolved topic in biology. Phylogenetic, developmental, and morphological approaches have led to competing theories regarding eye homology and evolutionary trajectories in each lineage [13–15]. The availability of transcriptomics and gene expression techniques for non-model arthropods is a boon to this topic and have been used to find homology in eye development and phototransduction in several arthropod taxa [16–19]. A recent project comparing eye morphology in two populations of isopods—one cave and one surface population—used QTL analysis combined with a candidate gene approach to map loci associated with both eye and pigmentation loss [20]. Sarsielloid ostracod crustaceans (Myodocopida) provide another approach to comparing development of eye types. Several species of sarsielloids are sexually dimorphic—males have large image-forming eyes while females have only rudimentary eyes that lack ommatidia. While QTL analysis would be difficult on these organisms, there is a published transcriptome for *Euphilomedes carcharodonta* juvenile male eyes from which candidate dimorphically expressed genes can be drawn [21]. *E. carcharodonta* eye dimorphism in particular is a promising subject for understanding the evolution, development, and genetics of compound eyes for several reasons.

First, the sarsielloid eye dimorphism is likely an example of an ecological sex–trait, rather than solely sexual selection, wherein males and females occupy different visual environments with different selective pressures [21]. Evidence for this was found in a previous predation study where male *Euphilomedes* were found to be predated on at a higher rate when their eyes were obscured, while female *Euphilomedes* were not [21]. Observational studies on related species find that males spend more time in the water column and females spend more time in the sand where they are not accessible to predators [22, 23]. Arguing against sexual selection driving the dimorphism is the finding that male visual acuity does not appear to allow for detection of females, sectioned adult male eyes have an interommatidial angle of 8° corresponding to a sensitivity of 2.3 µm^2^ ċsteradian [21]. Given these calculations, in bright clear water *Euphilomedes* males would be able to detect females at a range of 12 mm or less, while they would be able to detect juvenile fish predators (1 cm in length) at a range of 7.1 cm [21]. Together, these findings suggest a scenario where selection for image-forming eyes in males may be driven by their more predator-rich niche while selection against image-forming eyes, or neutral drift, in females may be driven by the higher energetic cost of building and maintaining eye tissue. Indeed, eye reduction has been found in many species living in light-poor environments, for example caves [24], and has been seen in particular in abyssal myodocopid ostracods [25, 26]. Thus, if eye degeneration is seen as the default state in the absence of visual data, the ecological significance of the different *Euphilomedes* eye types may mirror selective pressures driving the evolution of compound eyes. A similar scenario has been proposed for Lake Malawi cichlid fish, where craniofacial sexual dimorphism mirrors differences in ecomorphs. In both the fish and ostracods, the genetics underlying ecologically driven sexual dimorphism may very well also underlie variation between species [11].

**Fig. 1:**
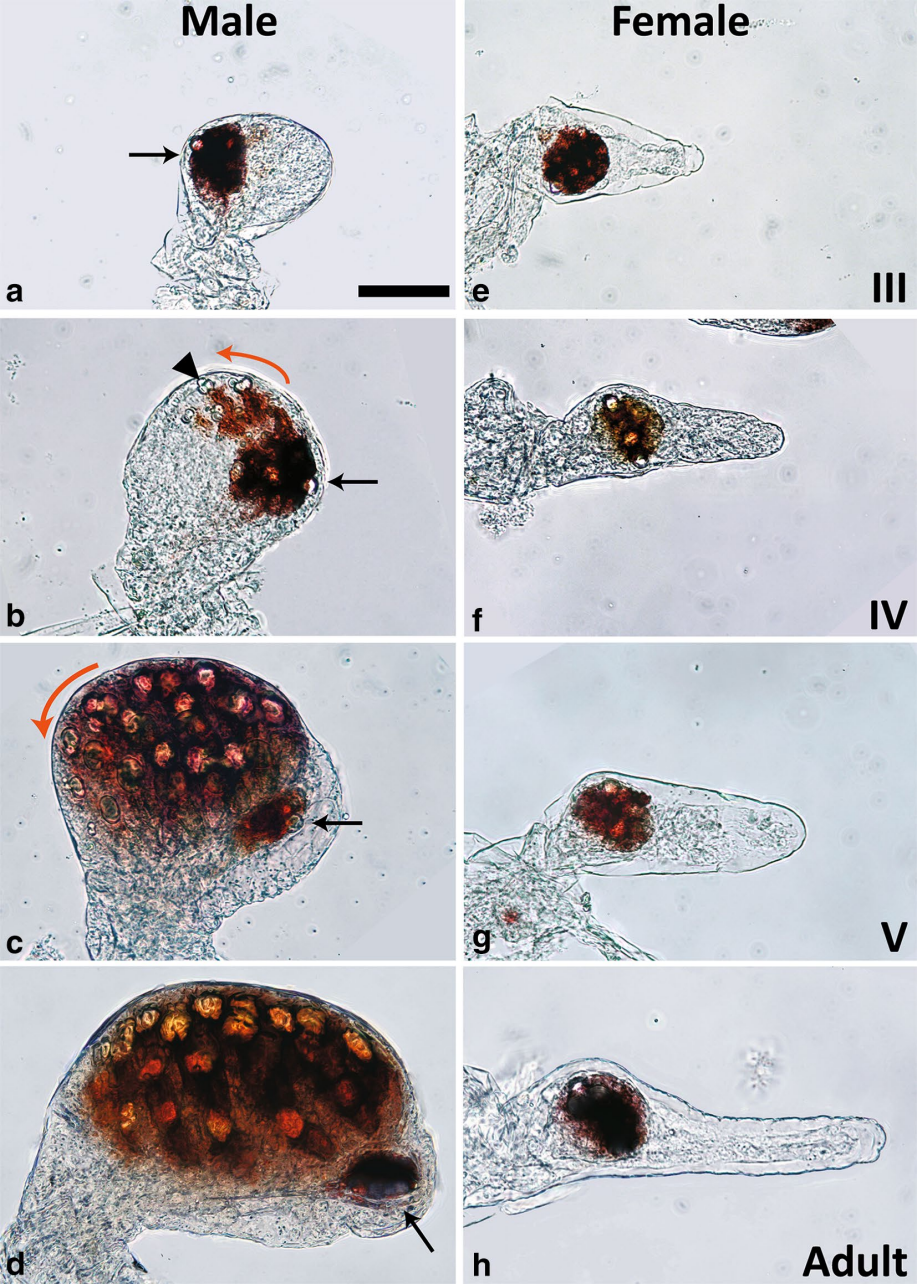
Eye ontogeny in *E. carcharodonta.* While male eyes undergo massive growth and differentiation during juvenile development (**a**–**d**), female eyes remain largely the same (**e**–**h**), Both male and female early juveniles have small stalked eyespots (**a**, **e**). Males and females retain these eyespots through adult stages (*arrows* in **a**–**d**), but males develop a secondary ommatidial field beginning at stage IV (**b**). Ommatidia are added in a distal to proximal direction (*orange arrows* in **b** and **c**) during stages IV and V. A developing ommatidium is marked with an *arrowhead* in **b** By adulthood, male eyes have over 30 ommatidia (**d**), while female eyes maintain their juvenile eyespot state (**h**). All images are scaled, *scale bar* in **a** is 50 μm

Second, the evolutionary history of dimorphism in sarsielloid ostracods may be complex with multiple losses and gains in different lineages. Studying dimorphism in one species will give us target genes to examine in studying the evolution of convergence. On a more practical note, eye differentiation happens externally during juvenile stages, making the eyes accessible for dissection and observation (Fig. 1). Finally, *Euphilomedes* has been shown to have XO/XX sex determination (as have other ostracod species) [27–30]. This eliminates the possibility that eye-development genes are present on a male-specific chromosome. Because of this, male/female differences in eye development must be regulated at the gene expression level, both morphs have near-identical genomic backgrounds. This makes *E. carcharodonta* an especially attractive model for studying the effects of gene expression differences on phenotype.

To study eye development and dimorphism in a non-model crustacean, we compare expression levels of several eye-related genes known to be involved in *Drosophila* eye development and phototransduction. Like many other animals that lack image-forming eyes, female *E. carcharodonta* retain rudimentary opsin-expressing eyes, suggesting that they express genes responsible for early eye-primordium specification, though they may not exhibit gene expression patterns associated with complex eye-field patterning. Most of what is known about the genetics of arthropod eye development comes from *Drosophila*, though similar patterning has been also seen in other pancrustaceans [31, 32].

In *Drosophila*, as is now known in many animals, eye specification begins with the PSED (or retinal determination) network [33]. This network consists of the core proteins Pax6, Sine Oculis (Six1/2), Eyes-absent, and Dachshund, as well as other developmental genes. Once the eye field has been specified, *Drosophila* use determination and patterning genes to pattern the eye into an array of ommatidia, each consisting of a conserved pattern of cells [34]. The first sign of patterning in a naïve eye is the expression of Hedgehog (Hh) and Hedgehog targets at the posterior margin of the eye imaginal disc [35]. This initiates the differentiation of the ommatidial cell types, beginning with photoreceptive cell R8 [31]. Hh induces the expression of Atonal (Ato) in the R8 cell, which in turn induces the expression of the secreted EGFR ligand Spitz (Spi) in the R8 cells. The secretion of Spi from R8, activates EGFR signaling in the surrounding cells [36–38]. The combination of this and Hh signaling induces the expression of Daughterless (Da) in cells adjacent to R8. Da, in combination with Notch-pathway lateral inhibition, prevents these other cells from assuming the R8 fate by negatively regulating Ato expression in these cells [39–42]. The combination of these factors leads to the sequential differentiation of the other seven photoreceptor cells followed by the differentiation of cone cells and pigment cells. The patterning of the final photoreceptor cell, R7, is particularly well studied as its determination is dependent on the conserved Sevenless/Ras1 pathway, including the E3 ligase Seven-in-absentia (Sina) [43].

Once major cell-fate decisions have been made, the cells themselves must undergo massive structural and gene expression pattern changes to differentiate into neural photoreceptor cells or structural cone and pigment cells. This requires the re-expression of some of the earlier genes. For example, EGFR is expressed in differentiating cone cells and induces neighboring cells to take on a pigment cell fate. EGFR activates a second Pax gene, Pax2/5/8 homolog Shaven, which is required for both the specification of cone and pigment cells as well as the specification of the sensory organ precursor cells that give rise to eye bristles [44, 45]. EGFR expression is required in another context, along with Dac, for neuronal differentiation in the laminar cells, which connect the CNS to the photoreceptor cells [46]. This differentiation also requires other genes, for example Elav, in a subset of the laminar cells. Elav is a pan-neuronal protein and thus required for the development and maintenance of R-cells and other neural cells in the ommatidium [47]. The photoreceptive structure in an ommatidium, the rhabdom, is made up of the extensive microvillar surfaces of the R-cells. The packing of these R-cells is key to the function of the ommatidium as a photosensory organ and is regulated, in part, by the glycoprotein Chaoptin (the protein product of *chaoptic*) [48, 49].

Whether an eye is complex or simple, to transduce a light signal into an electrical neuronal signal, the phototransduction cascade must be activated. In animals, the phototransduction cascade begins with the activation of Opsin [50, 51]. Light energy causes a change in Opsin conformation, which, in the rhabdomeric eyes of arthropods, activates Phospholipase-C via G protein signaling [52, 53]. This leads to an efflux of calcium through TRP channels, which triggers a cascade of protein activation leading to graded membrane depolarization, a transmissible electrical signal [54, 55]. This process is under tight control and the phototransduction cascade is quickly quenched and its effects reversed by Protein Kinase C (PKC), Calx, and Arrestin [56–58].

These *Drosophila* developmental and phototransduction proteins are candidates for differential expression in the dimorphic eyes of male and female ostracods. Here we examine the temporal expression of 13 genes in the developing eyes of *E. carcharodonta* males and females. We find that a homolog of the early patterning gene *dachshund* is expressed at higher levels in male eyes as are homologs of later patterning genes *shaven* and *chaoptic*. Not surprisingly, homologs of phototransduction genes *opsin* and *egfr* are expressed most highly in adult male eyes.

## Methods

### Collection of ostracods and eye dissections

No vertebrate animals were used in this study. Invertebrates were collected using a Field Permit granted to ASR from the California Department of Fish and Wildlife.

We collected *Euphilomedes carcharodonta* using hand-nets and sieves at Pillar Point, California (+37°29′ 55.22″, −122° 29′44.14″). During each collecting trip 2–4 researchers would wade out at low tide 50–100 m from shore, depending on kelp and tide, and use hand-nets to scoop sand from the top 1–2 cm of ocean floor. We then sieved the sand on shore to remove most of the sediment. We put the animals plus remaining sediment in buckets with sea water and cold packs for the 2–3 h trip back to the lab. In the lab, we kept the animals at 12–14 °C in plastic buckets with bubblers in sediment or in petri dishes once they had been removed from sediment. Animal condition appeared to be uniform, as assessed by size and activity of the animals. In particular, animals of the same stage were always within a tight size range.

We dissected live animals under a dissecting microscope, first sexing and staging the animals using the system outlined in Additional file 1. We then dissected the eyes by severing the eye from the body at the eye stalk using size 1 insect pins. Immediately after dissection, we placed eyes in 800 μL Trizol (Life Technologies) and stored for up to 2 weeks at 4 °C. Storage did not significantly affect RNA yield or amount of housekeeping genes *ec*-*EF1a*, *ec*-*28S*, or *ec*-*Actin* or developmental genes *ec*-*elav* or *ec*-*so17* (Additional file 2). Eyes from the same sex and stage were pooled (10 animals per pool) to obtain enough RNA for qPCR.

We took images on a Zeiss Axioscope A1 with DIC using a Canon EOS Rebel XS (Canon) and the remote camera function in EOS Utility software. We deconvolved image stacks using Helicon Focus software (HeliconSoft). We used Photoshop and Illustrator (Adobe) to adjust levels and montage images for figures.

### RNA isolation and generation of cDNA

Once we had 20 eyes from a particular sex and stage in a single tube of Trizol, we extracted RNA using modified manufacturer’s protocols. Briefly, we added 5 uL of glycogen and 160 uL of chloroform to the Trizol + eyes, shook for 20 s and incubated at room temperature for 5 min. We then centrifuged at 13,200 rpm in a 5430 centrifuge (Eppendorf) for 15 min at 4 °C. We removed the aqueous layer and added an equal volume of isopropanol and 1/10 volume of Sodium Acetate to it. We then precipitated overnight at −20 °C, centrifuged for 15 min at 4 °C and decanted the supernatant. We washed the resultant pellet in 1 mL of cold 75 % ethanol and centrifuged for 10 min at 4 °C. We removed the supernatant and let the pellet air dry for up to 10 min, then added 30uL of DEPC-treated water. We removed any DNA contamination using the Turbo DNAse Kit (Invitrogen) per the manufacturer’s instructions. We then quantified RNA with a Qubit Fluorometer (Life Technologies) using manufacturer’s protocols for the Broad Range RNA Detection kit. We used 40 ng of RNA in a 20 μL reverse transcriptase reaction using the SuperScript III Kit and Random Hexamers (Life Technologies). We used all 20 μL of cDNA immediately in qPCRs.

### Generation of phylogenetic hypotheses

Using *Drosophila* sequences for the genes *calx*, *six*-*1/2* (*sine*-*oculis*), *embryonic*-*lethal*-*abnormal*-*vision* (*elav*), *daughterless (da)*, *chaoptic (chp)*, *seven*-*in*-*absentia (sina)*, and *shaven (sv)* as baits, we performed blast searches against the nucleotide and protein translations of the *Euphilomedes carcharodonta* juvenile male eye transcriptome [21] locally with blast + using blastp and blastx searches [59]. In parallel, we used the *Drosophila* bait sequences to search the UniProt online database UniRef50 [60]. Using the top 50 hits from this search, our *E. carcharodonta* hits and our *Drosophila* baits, we created alignments with MUSCLE [61]. We then used a JTT model of evolution with PhyML, implemented in Seaview [62, 63] to build phylogenetic hypotheses (Additional file 3). We considered an *E. carcharodonta* genetic sequence a homolog to the group of interest if it fell within an in-group defined by previously annotated sequences. Homology was previously assessed using PIA (Phylogenetically Informed Annotation) for *E. carcharodonta* members of the *dachshund (dac)*, *epidermal*-*growth*-*factor*-*receptor* (*egfr*), *opsin (ops)*, *pax*-*6*, *phospholipase*-*C* (*plc*), and *proteinkinase*-*c* (*pkc*) gene families [21, 64].

### Plasmids for qPCR standard curves

Using the published transcriptome and annotations of *Euphilomedes carcharodonta*, our model in this study [21], we used Primer3 [65] to design primers against 13 genes. These primers were synthesized by Integrated DNA Technologies and are listed in Additional file 4.

We used the following cycling parameters: 95 °C for 5 min denaturation, followed by 92 °C for 45 s, 50 °C for 1 min, 72 °C for 1 min 30 s for 40 cycles using GoTaq Master Mix (Promega). We ligated these PCR products into pGEM-T Easy (Promega) and transformed into either TOP10 or DH5alpha competent cells following manufacturers’ protocols. We purified plasmids using QIAprep Spin Miniprep Kit (Qiagen) and the manufacturer’s protocols. Plasmids were sequenced by Sequetech (Mountain View, CA) and analyzed with CLC Bio Workbench (CLC Bio) software.

### qPCRs and statistics

To make standard curves for qPCR, we used three plasmid standards per gene at 1:10 dilutions, starting with 0.1–200 pg/µL. For each qPCR, we used 2 µL of cDNA or plasmid standards, 0.4 µL of the forward qPCR primer, 0.4 µL of the reverse qPCR primer (Additional file 4), 10 µL of SybrGreen (Invitrogen), and 7.2 µL of water. We ran the reaction using the Opticon Monitor 2 (BioRad) with the following program: 94 °C for 5 min and, repeated 40 times: 92 °C for 40 s, 52 °C for 1 min, 72 °C for 30 s, plate read, followed by a final melting curve protocol. We discarded runs with low efficiency (<80 %) or a low R2 value (<0.98). We set thresholds to discount any samples that went into exponential phase after cycle 35. The last set of cycles was to amplify any spurious secondary PCR products for detection in Melting Temperature analysis. Any primer sets that gave secondary PCR products were discarded. For the remaining reactions, we calculated values per reaction against each standard curve for each gene of interest and normalized with NORMA-Gene [66]. To test the efficacy of NORMA-Gene, we ran samples using housekeeping gene Actin for normalization and compared to NORMA-Gene levels, the expression levels were similar between the two methods of analysis (Additional file 5).

Statistical analysis on qPCR data was performed using R version 3.1.2 [67]. To assess gene expression differences across juvenile and adult stages and sexes, we employed two-way ANOVA tests. Since our sample sizes were small, ranging from 3 to 11 trials per gene-sex-stage combination, and expression levels differed across several orders of magnitude, we applied a Box-Cox transformation to the gene expression data prior to running each ANOVA to reduce skewness and heteroscedasticity between groups [68]. Optimal values of lambda were estimated to within 0.01 of their true values using the MASS package in R [69]. Note that since such transformations require strictly positive data, we replaced three observations recorded as 0 with one–half the minimum expression level in our sample prior to running the analysis. Post hoc analysis of pairwise comparisons of all sex/stage combinations and embryo/stage combinations were performed using Tukey’s Honest Significant Difference method, which adjusts for multiple comparisons. All results with *p* value <0.05 were reported as significant.

## Results

Male and female *Euphilomedes* ostracods exhibit extreme dimorphism in their eye morphologies although they have nearly identical genetic backgrounds. To study the genetic underpinnings of this dimorphic trait, we first identified candidate regulators of eye development from the published *E. carcharodonta* developing lateral eye transcriptome [21], then we compared gene expression levels between males and females across developmental stages.

### Eye development in *E. carcharodonta*

Male and female adult *E. carcharodonta* exhibit extremely dimorphic eye phenotypes, but their embryonic and early juvenile development is identical at the gross morphological level. While non-dimorphic myodocopid ostracods exhibit ommatidial development in embryos and maintain these compound eyes throughout development [70], *E. carcharodonta*, like another dimorphic species *E. morini*, have only simple eyes until midway through juvenile development, when males begin to form ommatidia [30, Fig. 1]. Instar IV marks the first visible eye dimorphism. At this stage, small lens cells associated with cones of pigment are visible in the male eyes, indicating ommatidial development (Fig. 1b, arrowhead). Lenses begin to form at the distal end of what will become the ommatidial field of the eye. A darkly pigmented tissue at the distal-most region of the eye field remains free of visible ommatidia [30, arrow Fig. 1b]. At instar V, males have an extensive ommatidial field with over a dozen large well-formed ommatidia (Fig. 1c). Adult males exhibit completely developed ommatidia but maintain the ommatidia-free region at the distal tip (arrow, Fig. 1d). Females, on the other hand, maintain only the small ommatidia-free tissue. Often a couple small lens-like cells are apparent, but these are not organized and not associated with an ommatidium (Fig. 1e–h).

### Eye-related genes from the *E. carcharodonta* transcriptome

Since *Euphilomedes* eye development is morphologically very similar to *Drosophila* eye development [30, Fig. 1], we searched for homologs of *Drosophila* eye-development genes in the *E. carcharodonta* transcriptome. Briefly, we retrieved *Drosophila* protein sequences from GenBank and used them as a query in blast searches of the *E. carcharodonta* transcriptome and the UniProt UniRef50 database [60]. We aligned the resultant hits with MUSCLE and generated Maximum Likelihood trees using PhyML implemented in Seaview [62]. We designed primers against and cloned *E. carcharodonta* sequences that fell within the in-group.

We dissected eyes from two developmental stages as well as from adult animals, extracted RNA using a standard Trizol protocol, and used 40 ng of total RNA in an RT-PCR to account for differences in starting tissue amounts. We then performed qPCR using Sybr chemistry, all reactions were performed in duplicate (see Additional file 4 for primer sequences). We quantified against plasmid standards of known quantity, using the same primer pairs for cDNA and purified plasmids. We normalized these quantities using NormaGene [66].

Using ANOVA and pairwise tests corrected for multiple comparisons [68, 69] we compared the expression levels of genes across stages and sexes. Upon doing this, we have found significant differences between males and females in a handful of eye-development genes. In addition, we have found stage-specific differences in eye gene expression. Taken together, this paints a profile of eye development at the genetic level for these extremely dimorphic eyes.

### Embryonic expression

**Fig. 2:**
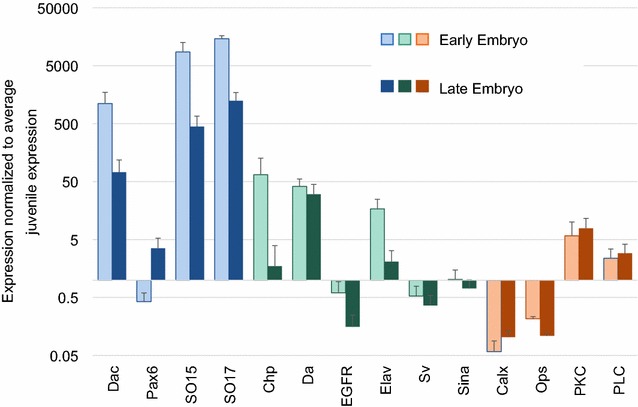
Embryonic expression of eye-related genes. To assess whether eye-development genes were also pleiotropically expressed at embryonic stages, we compared embryonic expression levels to average juvenile values. We divided average embryonic expression by average juvenile expression to assess relative expression levels. Numbers lower than one (below the X-axis) represent embryonic expression levels lower than average juvenile levels. Numbers greater than one (above the X-axis) represent embryonic expression levels higher than average juvenile levels. Most of the genes whose homologues are known to be used in *Drosophila* development, are also expressed at relatively high levels in *E. carcharodonta* embryos. Error bars represent standard error. Specification gene homologs are in blue, determination/patterning gene homologs are in green, and phototransduction gene homologs are in orange. The *lighter color* bars represent early embryos (no eyespot visible) while the *darker bars* denote late embryos (eyespot visible). Due to the large variation in expression levels between genes, the Y-axis is log-scaled

We found that most of the 13 eye-related genes expressed in male juvenile eyes are also expressed to some extent in embryos (Fig. 2). To further compare these values, we performed ratio of means and pairwise Tukey’s tests on embryonic versus juvenile and adult expression levels (Additional file 6). We found that developmental gene homologs *sine*-*oculis* (*ec*-*so17*) and *daughterless* (*ec*-*da*) were expressed at significantly higher levels in at least one embryonic stage than at any juvenile or adult sex or stage (Tukey’s test, p < 0.05). *dachshund* homolog *ec*-*dac* was expressed at significantly lower levels in late-juvenile and adult female eyes than in embryos (Tukey’s test, p < 0.05). While developmental genes *ec*-*egfr* and *shaven* homolog *ec*-*sv* were expressed at significantly lower levels in late-stage (eyespot visible) embryos compared to late-juvenile male eyes (Tukey’s test, p < 0.05). *Ec*-*egfr* was also expressed at higher levels in stage IV females than in late embryos (Tukey’s test; p < 0.05). Phototransduction genes *ec*-*calx*, *ec*-*opsin* (*ec*-*ops*), and *ec*-*plc*, as well as developmental gene *ec*-*pax6* were all expressed at significantly higher levels in adult males compared to both early (no eyespot visible) and late-stage embryos (Tukey’s test, p < 0.05). PLC expression was also significantly higher in adult females than in embryos (Tukey’s test, p < 0.05). Global comparisons of juvenile and embryonic expression patterns fell along the same lines (Fig. 2), with most developmental genes (blue and green) showing highest expression levels in embryos and phototransduction genes (orange) *ec*-*calx* and *ec*-*opsin* showing lower levels in embryos compared to juveniles. *Ec*-*pkc* and *ec*-*plc* were expressed at higher levels in embryos than juveniles.

### Genes without significant differences between the sexes

Nearly half of the eye-development and phototransduction genes examined did not have a significant difference between males and females in pairwise comparisons using the Box-Cox test (Additional file 7). These genes are from all levels of *Drosophila* eye development. Homologs of early acting genes (retinal determination network members) *ec*-*pax6* and *ec*-*so17* were expressed at similar levels in males and females at all stages examined, though *ec*-*so17* showed significant variation between biological replicates by ANOVA (Additional files 7, 8, 9). Likewise homologs of some genes acting later in *Drosophila* eye development, the specification and differentiation genes, also showed similar expression levels in males and females. These include *ec*-*da* and *ec*-*elav* (Additional files 7, 8, 9). A single phototransduction gene failed to show significant differences between males and females, *ec*-*pkc*. In this case, the ratio of means comparing adult male and female eyes was high, with average male expression nearly six times average female expression (Additional files 7, 9). However, the variability among biological replicates was high and pairwise comparisons between sexes/stages did not yield significance. Like *ec*-*so17*, *ec*-*pkc* also showed significant variation via ANOVA (Additional file 8).

### Genes with juvenile differences between male and female eyes

**Fig. 3:**
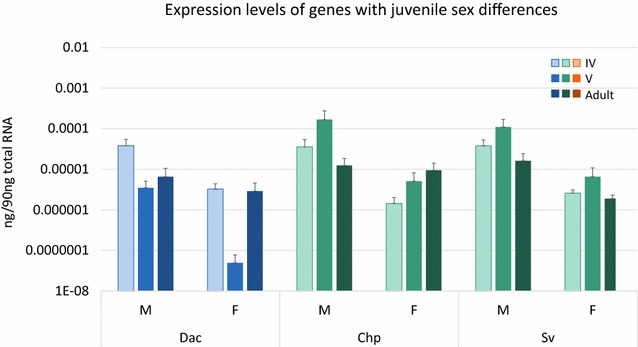
Genes with significant juvenile sex-specific differences in eye expression. Average quantities as determined by qPCR for genes with significant eye expression differences between stage-matched males and females. *Ec-dac*, *ec-chp*, and *ec-sv* all had significantly higher expression in juvenile males than in juvenile females. *ec-dac* and *ec-sv* showed the earliest differences, between stage IV male and female eyes. This difference was maintained in stage V males for *ec-sv*. *ec-chp* also showed significantly higher expression in male stage V eyes. All three genes showed significant variation by ANOVA (Additional file 9). *Error bars* represent standard error. Specification gene homologs are in *blue* and determination/patterning gene homologs are in *green*. *Lighter colors* denote earlier developmental timepoints. Due to the large variation in expression levels between genes, the Y-axis is log-scaled

Only three developmental gene homologs showed significant differences in expression between stage-matched developing male and female eyes. *ec*-*dac*, a homolog of a retinal determination pathway gene in *Drosophila*, showed significantly higher expression in male stage IV eyes compared to female stage IV eyes (Tukey’s test, p < 0.05; Fig. 3, Additional file 9). Stage V and adult female *ec*-*dac* eye expression levels were also significantly lower than male stage IV levels (Tukey’s test, p < 0.001). Homologs of *Drosophila* eye-determination/patterning genes *ec-sv* and *ec-chp* also showed significantly higher expression in male developing eyes compared to females. *Ec*-*chp* showed significantly higher expression in male stage V eyes compared to female stage IV and V (Tukey’s test, p < 0.01) while *ec*-*sv* was significantly higher in both male developmental stages compared to all female stages (Tukey’s test, p < 0.05; Fig. 3, Additional file 9).

### Genes with stage-specific differences

**Fig. 4:**
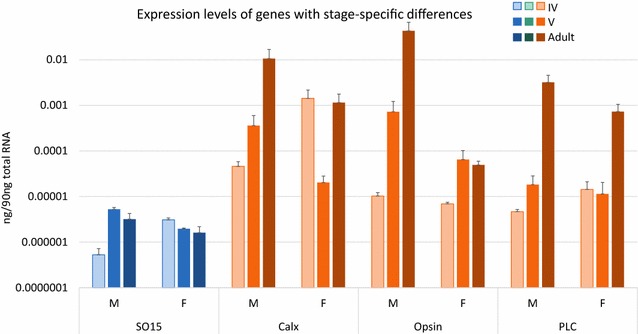
Genes with stage-specific differences. Average quantities as determined by qPCR for genes with significant eye expression differences between stages. *Ec-so15* showed higher expression in older male juveniles, when compared to younger male juveniles. Phototransduction genes *ec-calx*, *ec-ops*, and *ec-plc* all exhibited higher expression in adult males compared to at least two other stages. All four genes showed significant variation by ANOVA (Additional file 9). *Error bars* represent standard error. Specification gene homologs are in *blue*, and phototransduction gene homologs are in *orange*. *Lighter colors* denote earlier developmental timepoints. Due to the large variation in expression levels between genes, the Y-axis is log-scaled

Four genes exhibited significant expression differences between developmental stages within a sex. These were primarily phototransduction genes with high expression levels in adults. However, a single developmental gene, *ec*-*so15* showed significantly higher expression in stage V males compared to stage IV males (Tukey’s test, p < 0.05; Fig. 4, Additional file 9). Phototransduction gene homologs *ec*-*ops*, *ec*-*calx*, and *ec*-*plc* all had significantly higher expression levels in adult males when compared to other stages. *Ec*-*calx* showed higher adult male expression in pairwise comparisons against juvenile males (stage IV; Tukey’s test, p < 0.05) as well as juvenile females (stage V; Tukey’s test, p < 0.01; Fig. 4, Additional file 9). *Ec*-*ops* showed higher adult male expression when compared to all other sexes and stages (Tukey’s test, p < 0.05). *Ec*-*plc* showed higher adult male and female expression when compared to juveniles (Tukey’s test, p < 0.001; Fig. 4, Additional file 9).

## Conclusions

**Fig. 5:**
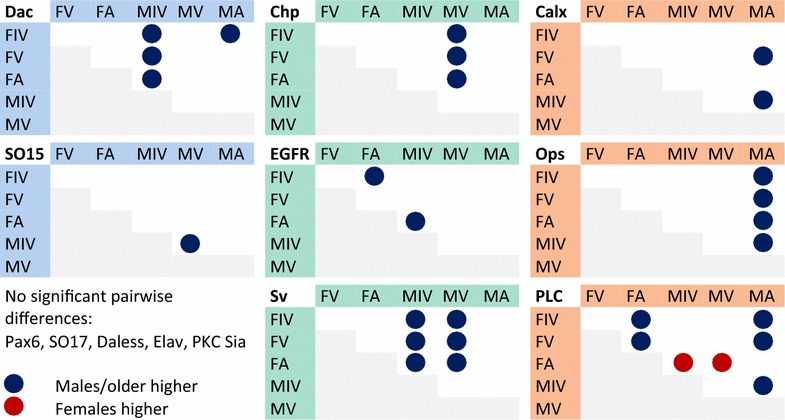
Summary of eye expression differences. We performed pairwise comparison of expression levels for 13 genetic loci at three developmental stages for both males and females. *Dots* indicate significant expression differences when adjusting for multiple comparisons. *Dark blue dots* indicate that either males (in male/female comparisons) or older developmental stages (in same-sex comparisons) expression levels were higher. *Red dots* indicate that female expression levels were higher. Homologs of early development (specification) genes are highlighted* blue*, later development (determination/patterning) are highlighted *green*, and phototransduction gene homologs are highlighted *orange*

Studying the genetic basis of polymorphism allows us to examine how a single, or similar, genotype can produce multiple phenotypes. This is the basis of developmental plasticity and can even allow us to predict the evolutionary history leading to complex morphologies [6, 10]. In this study, we used the extreme dimorphic eye phenotype in *E. carchardonta* ostracod crustaceans to begin to understand the evolution and development of crustacean eyes and sexually dimorphic traits. In *E. carcharodonta*, males exhibit large complex eyes with 33 ommatidia, likely capable of forming images [21], while females have only simple eyes and lack ommatidia and the ability to form images [30]. We compared the lateral-eye expression levels of genes known to act in arthropod eye development. Interestingly, males did not exhibit higher expression of all eye-development genes. Instead, only three of nine developmental genes showed higher expression in male developing eyes (Fig. 5). This suggests that similar genetic networks are used in the development of both compound and simple eyes in myodocopid ostracods. In addition, we find that eye-development genes are likely pleiotropic, used also during embryonic development, and that phototransduction genes are expressed at the highest levels in adult males (Fig. 6).

### *dachshund*, *shaven*, and *chaoptic* are expressed at higher levels in male developing eyes

Of nine developmental genes examined, only three showed significantly higher levels in male developing eyes compared to female developing eyes, *ec*-*dac*, *ec*-*sv*, and *ec*-*chp* (Figs. 3, 5, Additional file 9). All three of these genes play a role in compound eye patterning in *Drosophila*, though Dac also plays a role in early eye-field specification [34]. The later role of Dac, in eye patterning, involves differentiation of the neuronal cells connecting the photoreceptive eye cells to the central nervous system [46]. Sv, a Pax-2/5/8 homolog, is involved in specification of several different types of ommatidial cells including cone cells, pigment cells, and sensory organ precursor cells [44, 45]. Chp is involved in arranging the photoreceptive cells in the ommatidia to form a light guide [48, 49]. In *E. carcharodonta*, both *ec*-*dac* and *ec*-*sv* are expressed at higher levels in male eyes than female eyes at the earliest juvenile stages examined (Figs. 3, 5 and Additional file 9). At this stage, male eyes are just beginning to develop ommatidia (Fig. 1). As Dac and Sv are both transcription factors, it is likely that they are early regulators of the phenotypic difference between male and female eyes.

It is important to note that not all eye-development genes are upregulated in males, in fact similarity between male and female eye development is more common than difference. Moreover, since eye development occurs in a spatial gradient (Fig. 1), genes are not expressed in a strict temporal pattern over the whole eye. That is, specification genes and differentiation genes could be expressed in different eye regions at the same time. Future studies looking at fine scale in situ *hybridization* will allow us to further dissect this.

### Eye-development genes are pleiotropic

All of our genes were found in the male juvenile eye transcriptome, but our developmental genes are also known to play a role in other aspects of *Drosophila* development. To test whether our candidate eyes genes were pleiotropic developmental genes in *E. carcharodonta*, we compared expression in juvenile eyes to embryonic expression. We found that most developmental genes are expressed at higher levels, between 10- and 10,000-fold, in whole embryos compared to developing juvenile eyes, including *ec*-*dac* and *ec*-*chp* (Fig. 2, Additional file 9). This strongly suggests that most of the genes involved in *Drosophila* eye development are also used in other developmental contexts in *E. carcharodonta*. This finding is not surprising, since many developmental genes are highly pleiotropic. This also suggests that eye-development genes, including ones involved in complex eye organization, may be maintained even in the absence of compound eyes because they are necessary in multiple early developmental processes. This suggests that even if male eyes are only slightly advantageous, the genes specifying and organizing them could be maintained due to pleiotropy in embryonic development in both males and females.

### Most phototransduction genes are expressed at higher levels in male adult eyes

Of the four phototransduction genes examined, adult male eyes had significantly higher levels of expression compared to female eyes for three of them, *ec*-*calx*, *ec*-*ops*, and *ec*-*plc* (Figs. 4, 5, Additional file 9). This is not surprising since adult male eyes are ommatidial. Ommatidial eyes have much more photoreceptive membrane than simple eyespots and should theoretically express more proteins involved in phototransduction. Surprisingly, female eyes also expressed high levels of *ec*-*plc*, compared to earlier male and female stages (Fig. 5, Additional file 9). This, as well as the finding that females express all phototransduction genes (Fig. 4, Additional files 7, 9) suggests that adult females are using their eyes to sense light and may have a higher reliance on light cues than juveniles, perhaps due to resource acquisition or mating habits. Currently the relative light sensitivity of females and juvenile *Euphilomedes* is unknown. But the observation that females are expressing high levels of *ec*-*plc* suggests a series of future functional experiments comparing the sensitivity of different stages and sexes to light. Two behaviors that have been observed for *Euphilomedes* are burrowing in the sand and swimming. Testing these behaviors in different light regimes might show that adult females have a higher light sensitivity or faster response to light than juveniles.

### Differentially expressed genes represent possible regulators of evolutionary change

Male and female *Euphilomedes* ostracods have the same suite of developmental genes, yet have extremely dimorphic eye phenotypes. The differential regulation of a handful of eye-development genes may underlie this dimorphism. Since these genes are also likely used in other developmental contexts (Fig. 2), it stands to reason that their pleiotropy may help to maintain them even in environments that select against large image-forming eyes. Some lineages of myodocopid ostracods appear to have lost eyes when moving to deep-sea habitats [26]. Pleiotropy of eye-development genes suggests that these losses may occur via gene expression changes, rather than protein changes or loss of function mutations. A second possibility is that some of these genes have undergone duplication and subfunctionalization of their roles in eye development and general development. In our study, we found that the expression patterns of two *six*-*1/2* (*ec*-*so15* and *ec*-*so17*) homologs differ from each other in *E. carcharodonta* (Additional file 9). One copy (*ec*-*so17*) does not show significant differences during development of simple vs. ommatidial eyes, while the other copy (*ec*-*so15*) is expressed at significantly higher levels in developing ommatidial eyes. An analysis of the *E. carcharodonta* juvenile male transcriptome uncovered several eye-related gene families with multiple members [21], all of these are potential targets for differential regulation.

How are these genes expressed in different patterns in males and females? Genomic sequencing of the regions surrounding the eye-development genes in *E. carcharodonta* may uncover conserved cis-regulatory elements in genes with similar expression patterns. This would tell us whether the regulation of the dimorphically expressed genes is part of a regulatory cassette that evolved from existing expression patterns or whether each gene itself has evolved new regulatory regions that give it a dimorphic expression pattern in *Euphilomedes*. Comparing these to animals from other dimorphic and non-dimorphic ostracod lineages could tell us how the gene regulation has evolved—are female eyes turned off, or is an existing cassette turned on in males?

To further understand the development of this phenotype, candidate genes for dimorphism regulation could be examined at the expression level as well as via binding studies. For example, transcriptome analysis of male juvenile eyes uncovered a possible *E. carcharodontadoublesex* homologue, a component of the sex-determination pathway in a number of Metazoa [63]. Finding this in male eyes raises the intriguing possibility that this sex-determination gene is an upstream regulator of eye dimorphism. Cis-regulatory analysis, binding studies, and expression studies could all potentially shed light on this by elucidating *dsx* targets in *E. carcharodonta*.

### Sexual dimorphism and the evolution of complexity

This study builds on a field linking the genetics of sexual dimorphism to the genetics of macroevolutionary change. Previous work in butterflies showed that *optix* gene expression patterns in a dimorphic species mirror the expression changes that give wing pattern variation among members of the genus [9]. Similarly, both cichlid and stickleback fish show sexual dimorphism in craniofacial structures on par with differences seen in species occupying different ecological niches [10, 11]. Ostracod eye morphologies also seem to vary with ecology—large compound eyes are associated with shallow water habitats [25, 26]. On a larger scale, arthropod eyes in general are extremely variable, with an uncertain evolutionary history. If differences in gene expression can code the difference between a compound and simple eye in the same genetic background, this could begin to explain the complex pattern of simple and compound eyes seen in the Arthropoda and potentially help explain how this group exhibits such a large array of optical types [12].

## Additional files

10.1186/s13227-015-0026-2: Staging and sexing of *E. carcharodonta.* We sexed animals under a dissecting microscope by examining the morphology of the endopodite of the second antenna [1]. A (female, stage IV) and C (male, stage IV) show the position of the endopodite on the second antenna, with respect to the exopodite and protopodite. Female endopodites are pointed at the distal tip (B) while male endopodites (D) are rounded at the distal tip. In cases where there was ambiguity, we examined the second antenna under a compound scope. We staged the animals by examining the morphology of their furca [1]. We counted the proximal primary and secondary claws (E–G) to find the instar stage of the animal—adults have 6 proximal claws (E), instar Vs have 5 proximal claws (F), instar IVs have 4 (G).
10.1186/s13227-015-0026-2: Comparison of stored vs. fresh RNA. We compared qPCR results for ec-28S, ec-actin, ec-ef1a, ec-elav, and ec-so17 using fresh RNA or RNA stored for 1 month in Trizol at 4 °C from early embryos, late embryos, and female stage V eyes. We ran two trials and obtained similar values. We performed paired two-tailed Student’s t-tests comparing fresh vs. stored RNA and did not get significant differences when comparing all genes in a single test or when performing paired t-tests for each gene separately.
10.1186/s13227-015-0026-2: Maximum Likelihood trees. We built phylogenetic trees for Sine Oculis (Six 1/2), Elav, Seven-In-Absentia (Sia), Daughterless (Da), Shaven (Pax 2/5/8), Chaoptic, and Calx to determine which *Euphilomedes carcharodonta* transcriptome blast hits are members of these gene families. Other members were determined using PIA analysis [22].
10.1186/s13227-015-0026-2: Primers. Transcriptome or GenBank identifiers for each gene used in this study, type of phylogenetic hypothesis used to asses homology, and primers for cloning and qPCR are listed. Specification genes are in blue, Pattering/Differentiation genes are in green, and Phototransduction genes are in orange.
10.1186/s13227-015-0026-2: Comparison of normalization strategies. Using the same qPCR data from 3 housekeeping and 2 developmental genes, we normalized either using NormaGene software [62] or by normalizing to *Ec*-*actin* levels for the same pool of cDNA. We ran two trials with three developmental timepoints each, early embryos (ee), late embryos (le) and stage V females (FV). In trial 1, we used two separate RNA preparations of late embryos and in trial 2 we used two separate RNA preparations of stage V females. Expression patterns were similar between normalization strategies, though the values after Actin normalization are apparently higher since values are divided by *Ec*-*actin* pg values, which were always less than 1.
10.1186/s13227-015-0026-2: Ratio of means for early embryos. The ratio of means for early embryos vs. all other stages for each gene is shown here. Red numbers and pink numbers indicate genes/stages where Tukey’s test showed significantly higher expression in embryos. Black, blue, and purple numbers indicate genes/stages where Tukey’s test showed significantly lower stages in embryos. Specification genes are highlighted in blue, Determination/Patterning genes in green, and Phototransduction genes in orange.
10.1186/s13227-015-0026-2: Genes without significant sex/stage differences in eye expression. Average qPCR values for genes without significant differences in expression between the sexes. Ec-Pax-6, Ec-SO17, Ec-Da, Ec-Elav, and Ec-PKC did not show significant differences in pairwise comparisons of expression levels between sexes and stages. Error bars represent standard error. Asterisks highlight genes that showed significance in ANOVA, but not significant pairwise differences using Box-Cox tests. Specification gene homologs are in blue, determination/patterning gene homologs are in green, and phototransduction gene homologs are in orange. Lighter colors denote earlier developmental timepoints. Due to the large variation in expression levels between genes, the Y-axis is log-scaled.
10.1186/s13227-015-0026-2: ANOVA results. ANOVAs were run for the expression values for each gene and reported here. Red numbers indicate ANOVA p-values < 0.05. Asterisks indicate significant pairwise differences between at least one sex/stage by Box-Cox analysis. Specification genes are highlighted in blue, Determination/Patterning genes in green, and Phototransduction genes in orange.
10.1186/s13227-015-0026-2: Ratio of means for juvenile and adult stages. We report the results of ratio of means analysis here. Comparisons that were significant in pairwise Tukey’s t-tests (adjusted for multiple comparisons) are blue (p < 0.5), green (p < 0.01) or red (p < 0.001). Specification genes are highlighted in blue, Determination/Patterning genes in green, and Phototransduction genes in orange.

## Abbreviations

QTL: quantitative trait loci
PSED: pax/six/eya/dac
R-cell: rhabdomeric cell
*ec*: *Euphilomedes carcharodonta*
DEPC: diethylpyrocarbonate
qPCR: quantitative PCR
