# Supplementary figures and images for: Sexually dimorphic gene expression in the lateral eyes of Euphilomedes carcharodonta (Ostracoda, Pancrustacea)
Source: EvoDevo. 2015 Nov 10;6:34. doi: 10.1186/s13227-015-0026-2 (PMC4641368; doi:10.1186/s13227-015-0026-2)

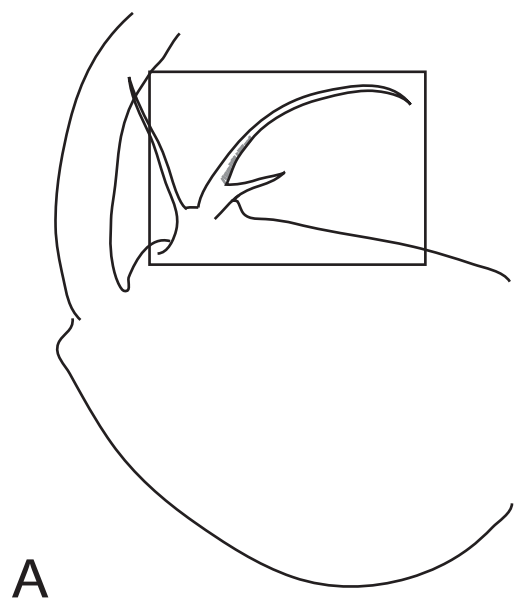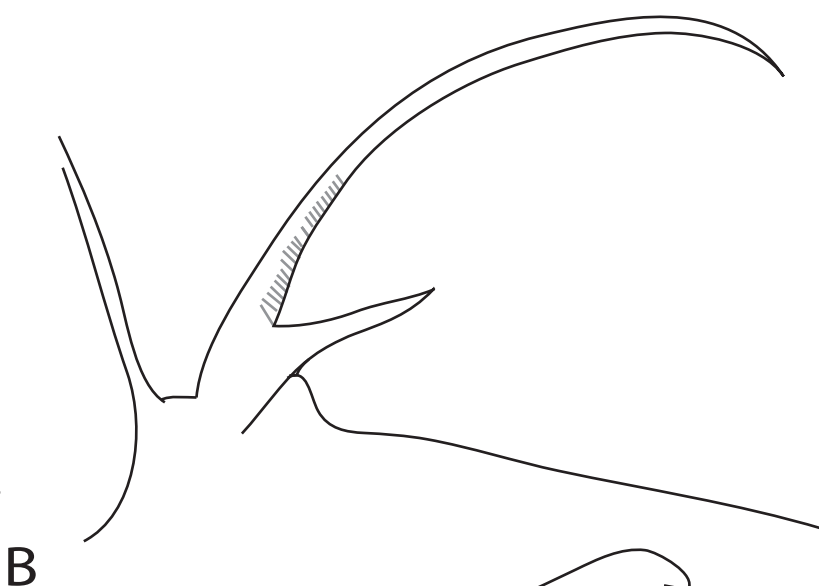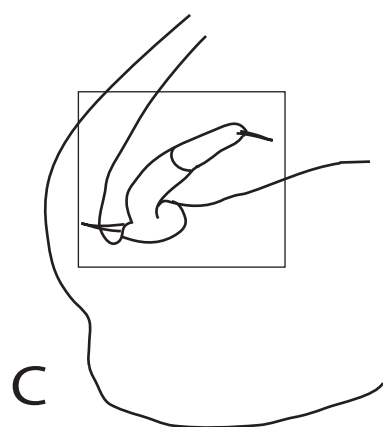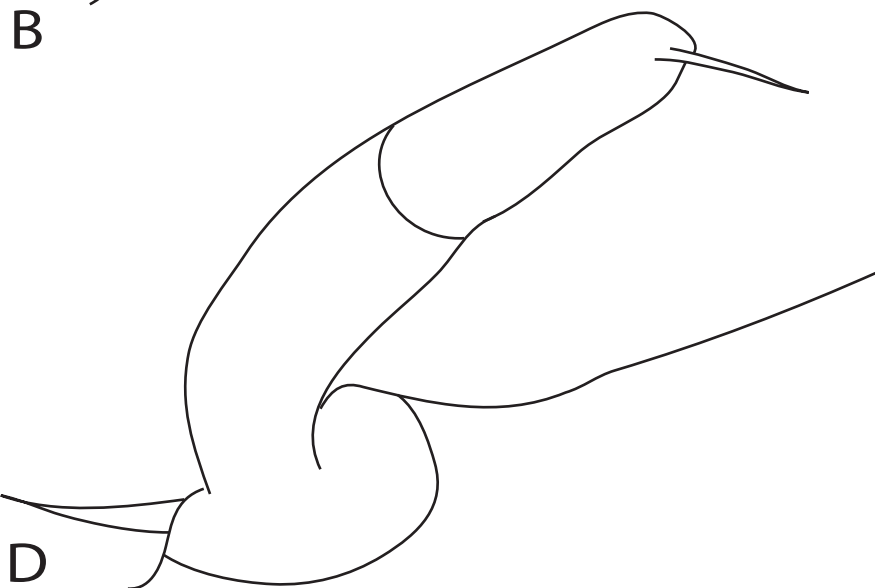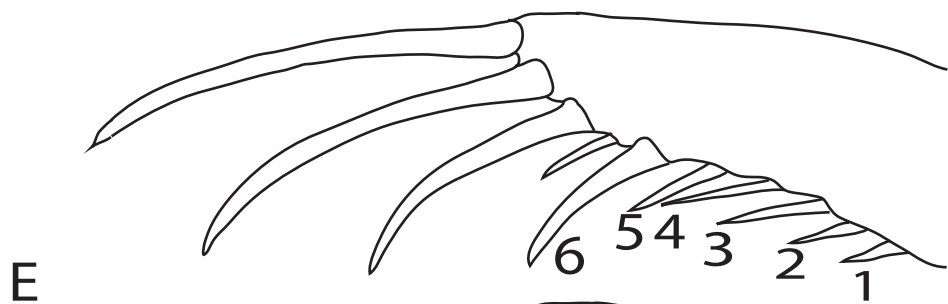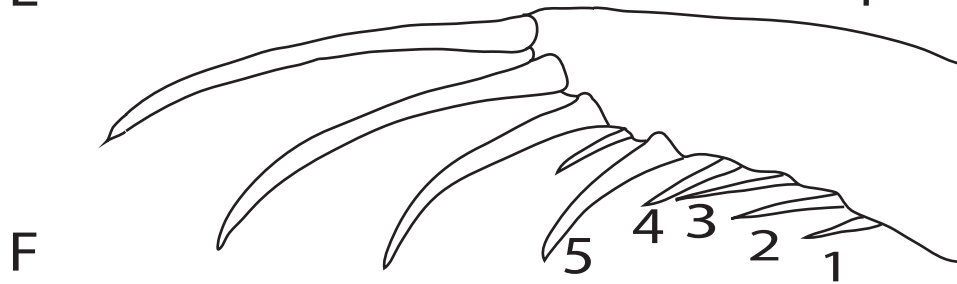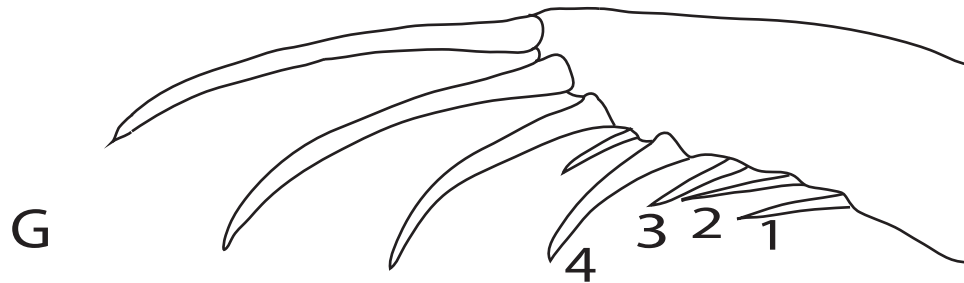

Supplement: Supplementary file 1 — 10.1186/s13227-015-0026-2: Staging and sexing of E. carcharodonta. We sexed animals under a dissecting microscope by examining the morphology of the endopodite of the second antenna [1]. A (female, stage IV) and C (male, stage IV) show the position of the endopodite on the second antenna, with respect to the exopodite and protopodite. Female endopodites are pointed at the distal tip (B) while male endopodites (D) are rounded at the distal tip. In cases where there was ambiguity, we examined the second antenna under a compound scope. We staged the animals by examining the morphology of their furca [1]. We counted the proximal primary and secondary claws (E–G) to find the instar stage of the animal—adults have 6 proximal claws (E), instar Vs have 5 proximal claws (F), instar IVs have 4 (G). [file 13227_2015_26_MOESM1_ESM.pdf]

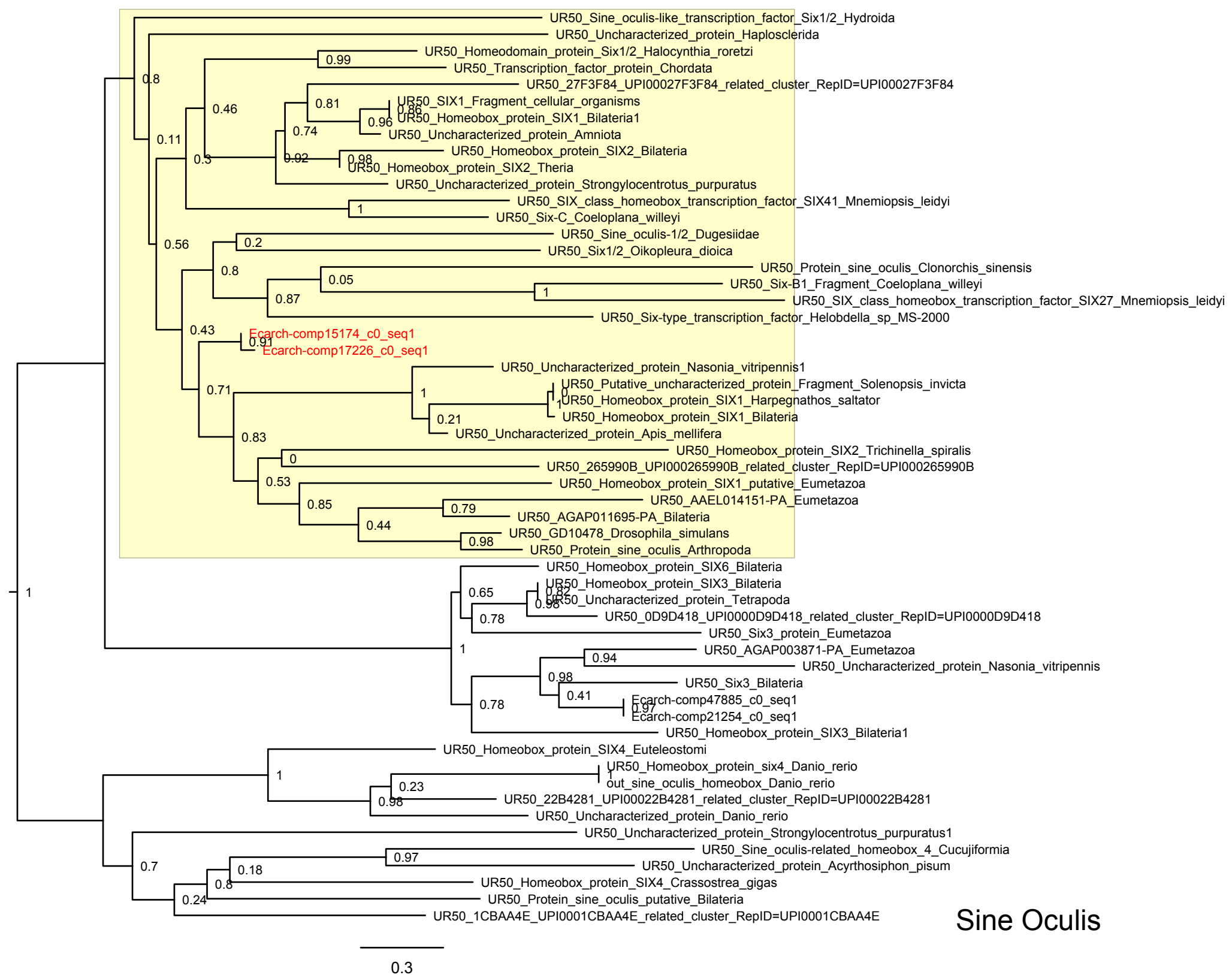

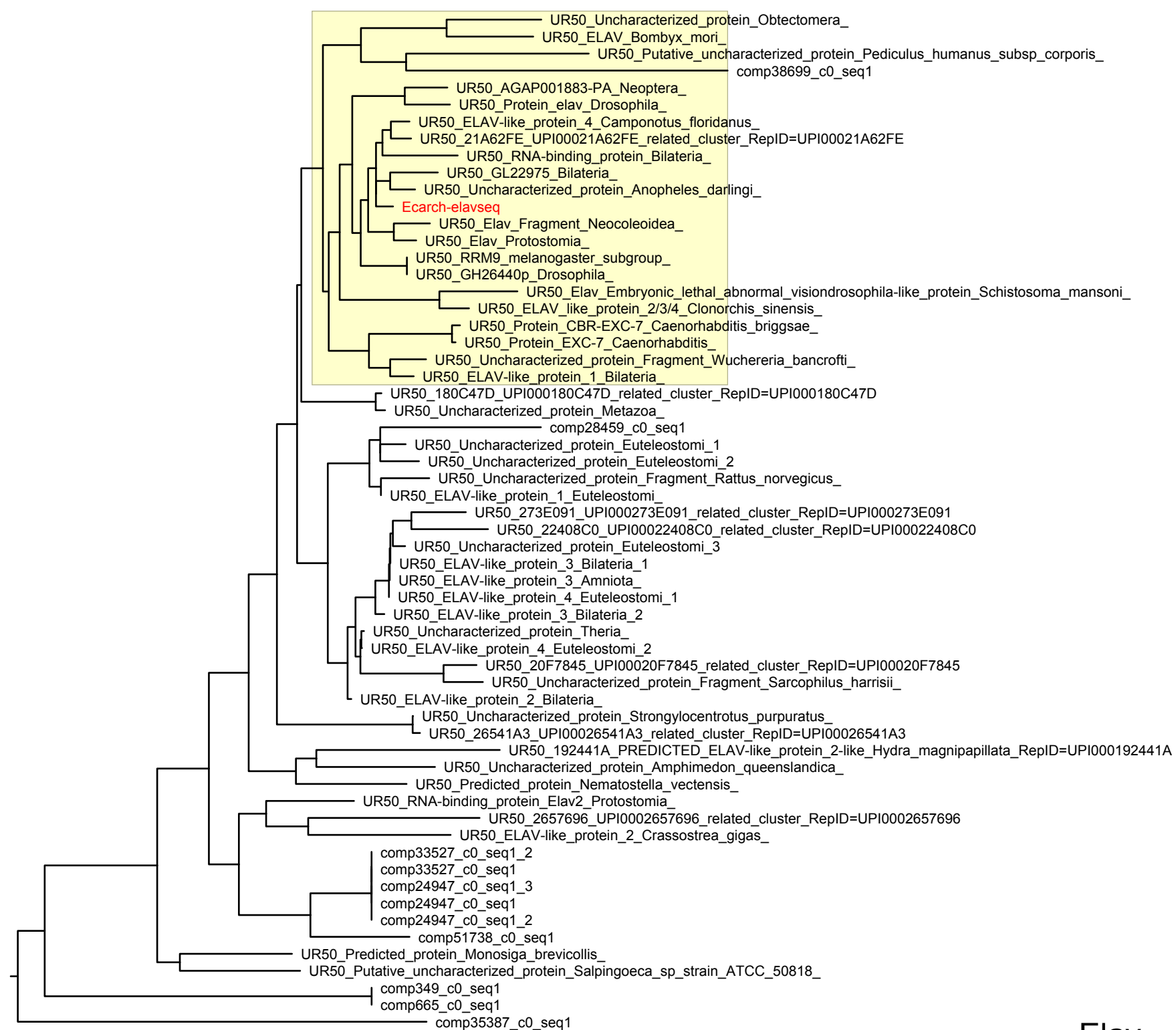

Elav

0.8

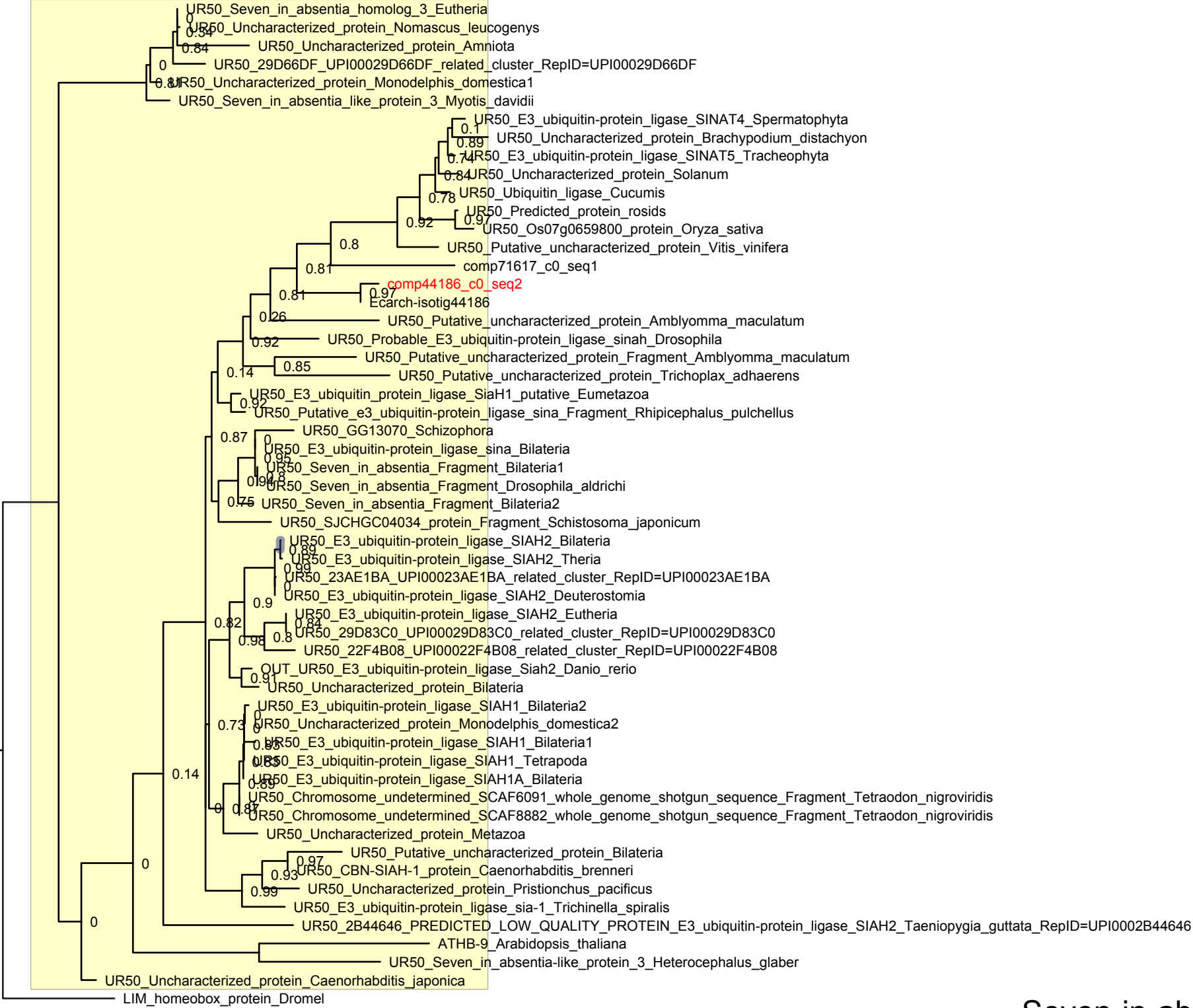

Seven-in-absentia

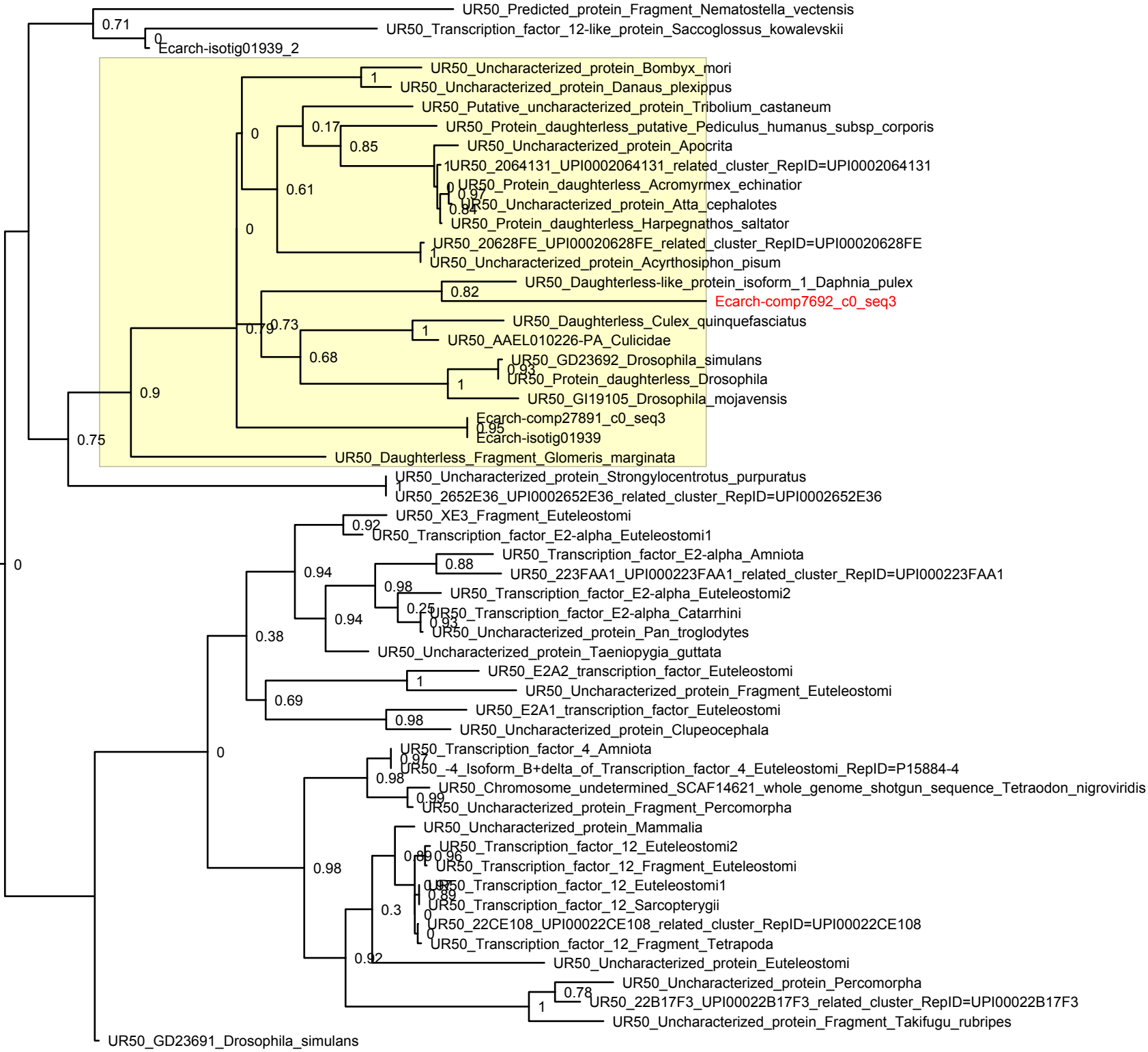

Daughterless

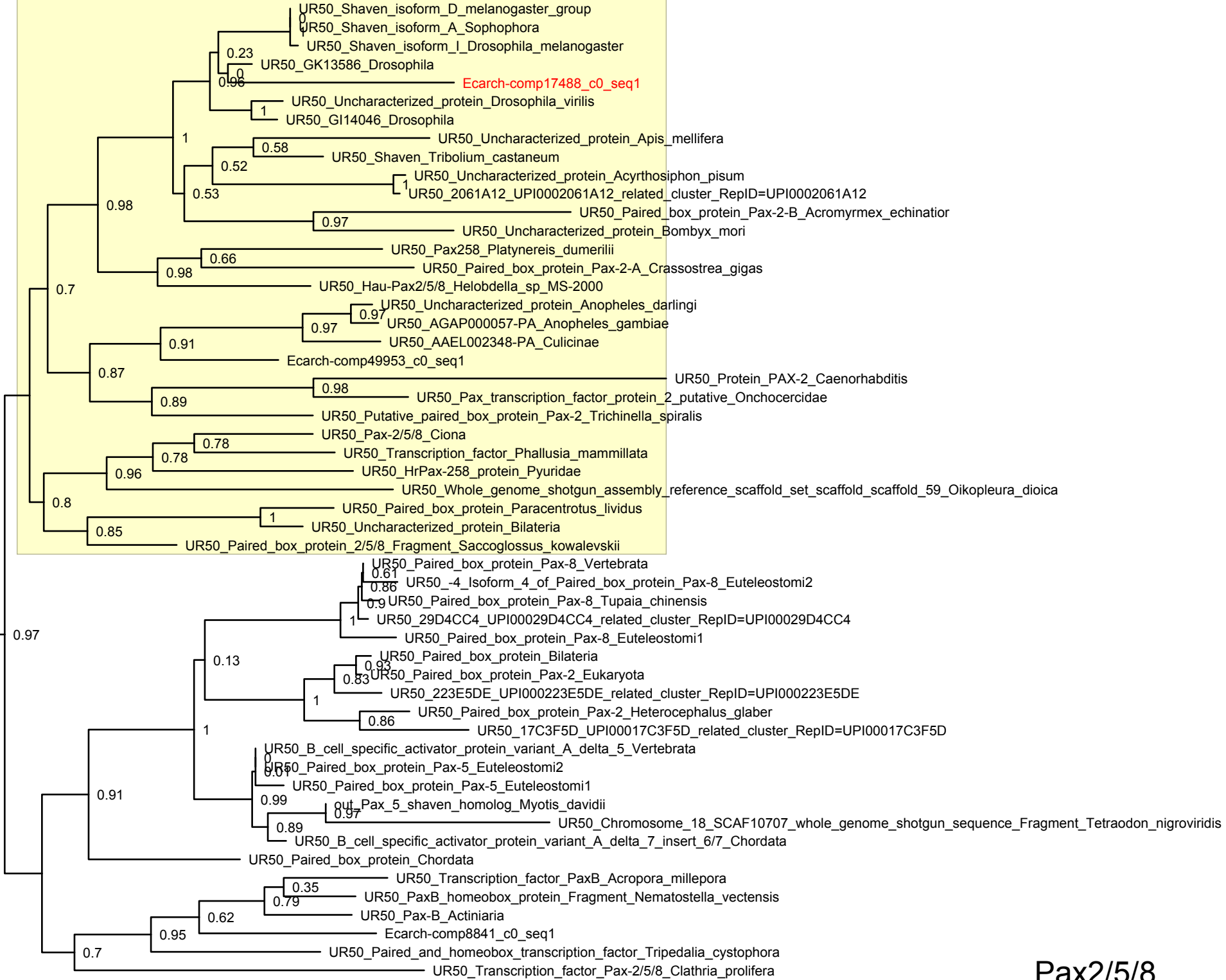

Pax2/5/8  
Shaven

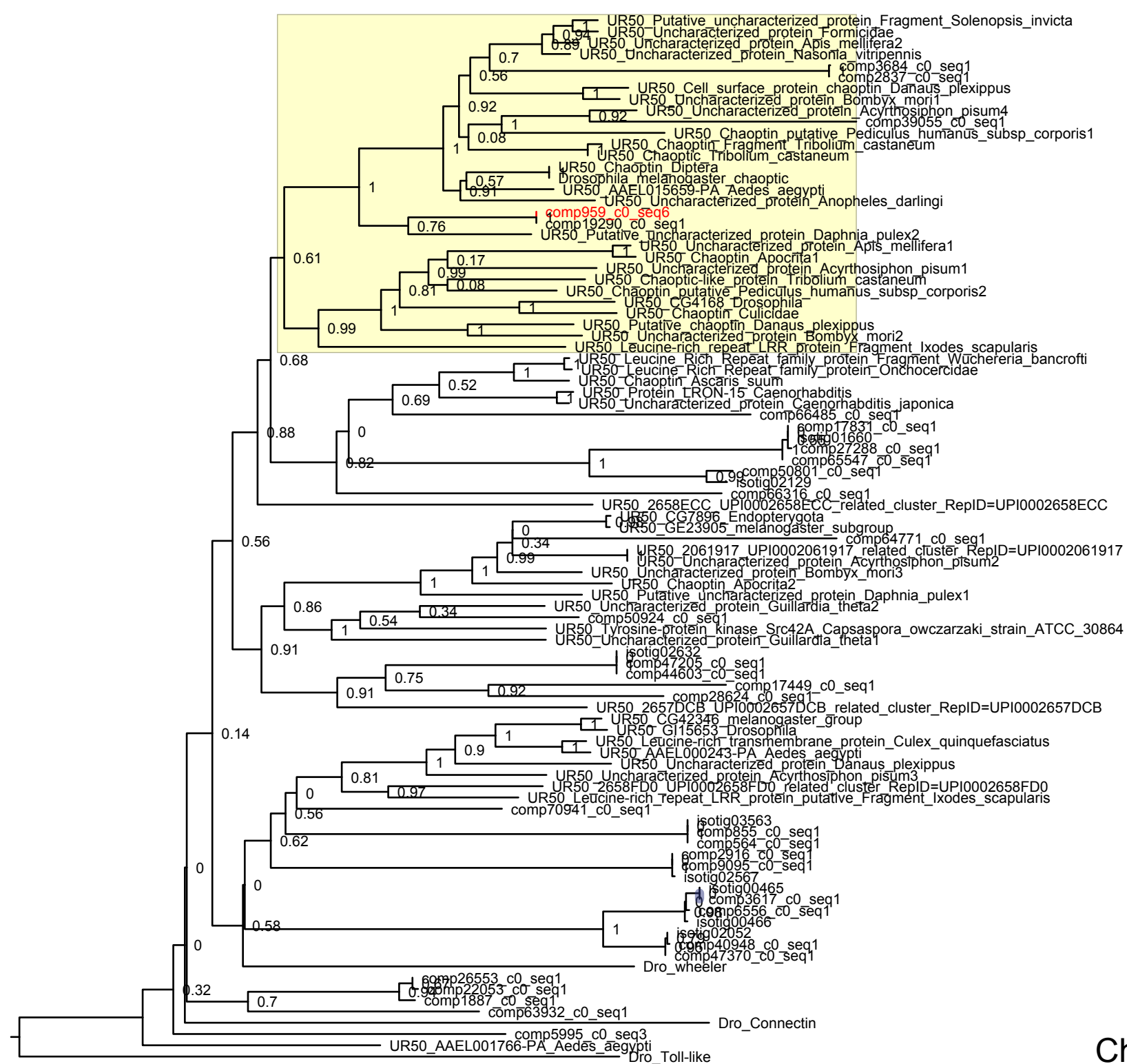

Chaoptic

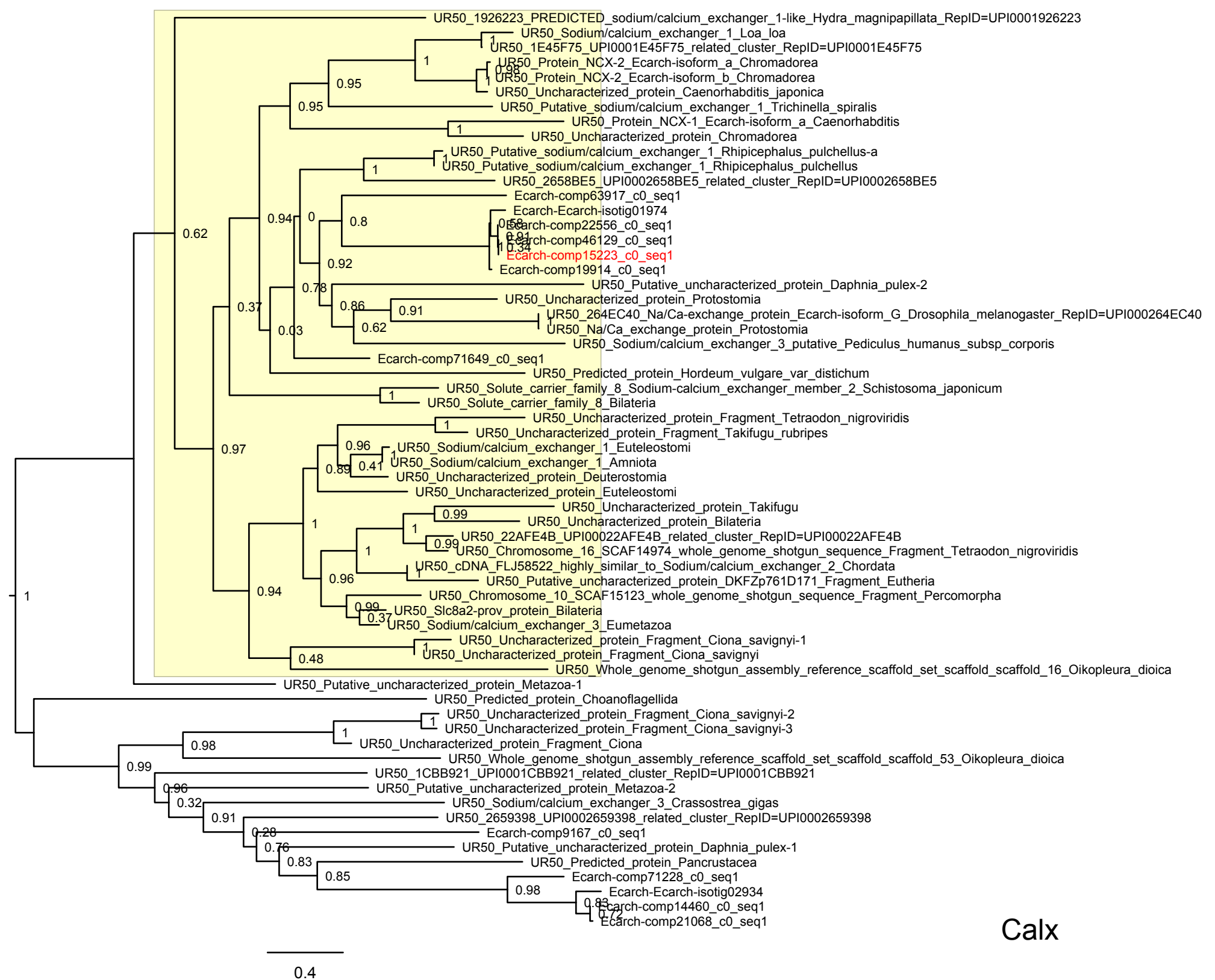

Supplement: Supplementary file 3 — 10.1186/s13227-015-0026-2: Maximum Likelihood trees. We built phylogenetic trees for Sine Oculis (Six 1/2), Elav, Seven-In-Absentia (Sia), Daughterless (Da), Shaven (Pax 2/5/8), Chaoptic, and Calx to determine which Euphilomedes carcharodonta transcriptome blast hits are members of these gene families. Other members were determined using PIA analysis [22]. [file 13227_2015_26_MOESM3_ESM.pdf]

Trial 1 - NormaGene

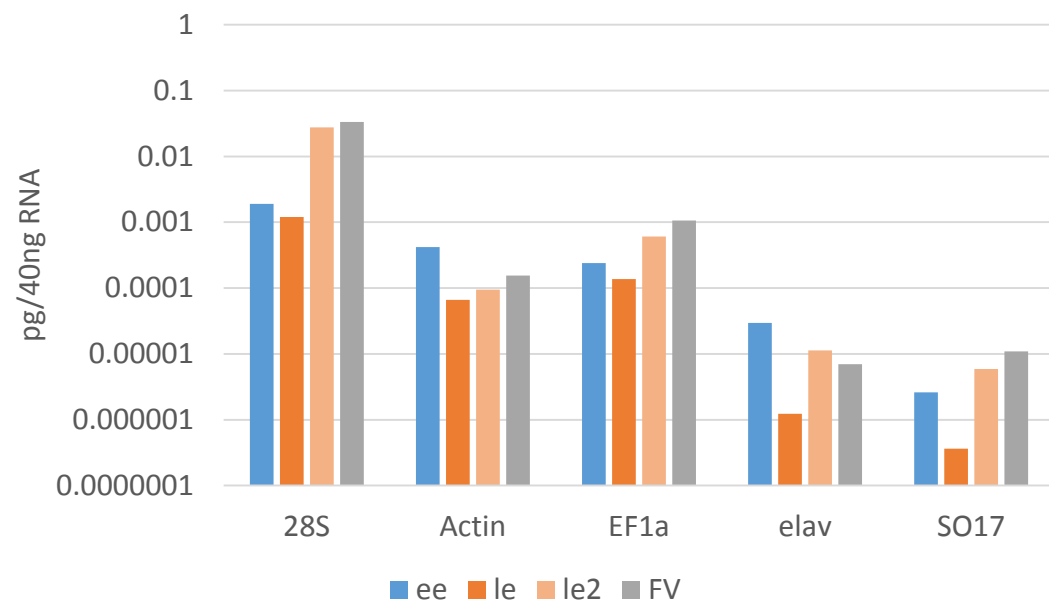

Trial 1 - Actin normalization

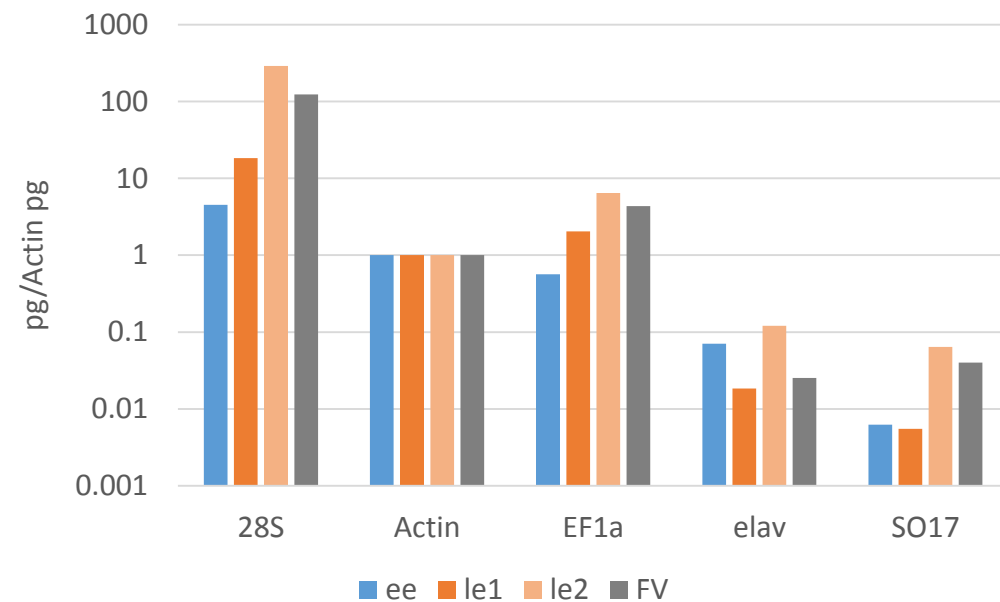

Trial 2 - NormaGene

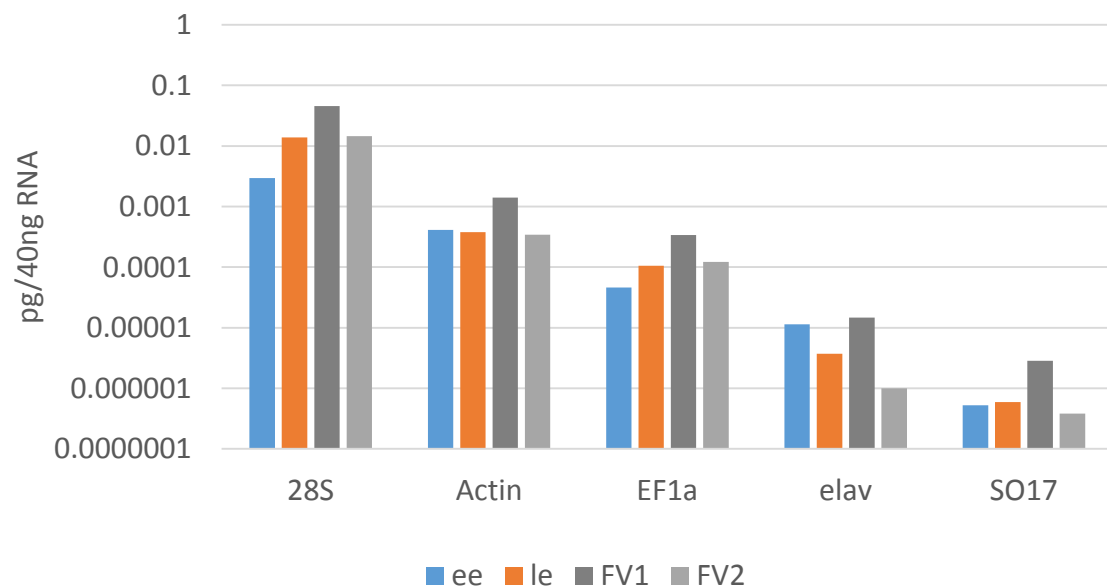

Trial 2 - Actin normalization

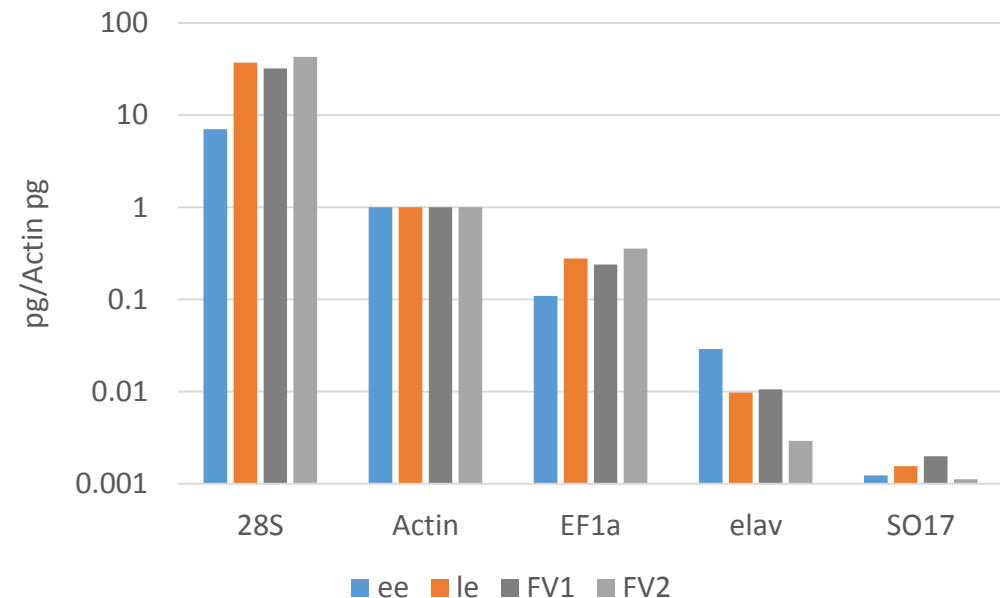

Supplement: Supplementary file 5 — 10.1186/s13227-015-0026-2: Comparison of normalization strategies. Using the same qPCR data from 3 housekeeping and 2 developmental genes, we normalized either using NormaGene software [62] or by normalizing to Ec-actin levels for the same pool of cDNA. We ran two trials with three developmental timepoints each, early embryos (ee), late embryos (le) and stage V females (FV). In trial 1, we used two separate RNA preparations of late embryos and in trial 2 we used two separate RNA preparations of stage V females. Expression patterns were similar between normalization strategies, though the values after Actin normalization are apparently higher since values are divided by Ec-actin pg values, which were always less than 1. [file 13227_2015_26_MOESM5_ESM.pdf]
