# Supplementary material for: Sexually dimorphic gene expression in the lateral eyes of Euphilomedes carcharodonta (Ostracoda, Pancrustacea)
Source: EvoDevo. 2015 Nov 10;6:34. doi: 10.1186/s13227-015-0026-2 (PMC4641368; doi:10.1186/s13227-015-0026-2)
Supplement: Supplementary file 2 — 10.1186/s13227-015-0026-2: Comparison of stored vs. fresh RNA. We compared qPCR results for ec-28S, ec-actin, ec-ef1a, ec-elav, and ec-so17 using fresh RNA or RNA stored for 1 month in Trizol at 4 °C from early embryos, late embryos, and female stage V eyes. We ran two trials and obtained similar values. We performed paired two-tailed Student’s t-tests comparing fresh vs. stored RNA and did not get significant differences when comparing all genes in a single test or when performing paired t-tests for each gene separately. [file 13227_2015_26_MOESM2_ESM.pdf]

# Comparisons in expression levels between fresh RNA and stored RNA (1 month in Trizol at 4 degrees C)

**paired t-test (two tailed)**
**between all stages and genes**
p=0.771938

**Paired t-tests for each gene**

| | |
|---|---|
| p=0.776621 | **28S** |
| p=0.256654 | **Actin** |
| p=0.155536 | **EF1a** |
| p=0.521777 | **elav** |
| p=0.169066 | **SO17** |

stages examined: early and late embryos, female stage V eyes
