# Supplementary material for: Sexually dimorphic gene expression in the lateral eyes of Euphilomedes carcharodonta (Ostracoda, Pancrustacea)
Source: EvoDevo. 2015 Nov 10;6:34. doi: 10.1186/s13227-015-0026-2 (PMC4641368; doi:10.1186/s13227-015-0026-2)
Supplement: Supplementary file 4 — 10.1186/s13227-015-0026-2: Primers. Transcriptome or GenBank identifiers for each gene used in this study, type of phylogenetic hypothesis used to asses homology, and primers for cloning and qPCR are listed. Specification genes are in blue, Pattering/Differentiation genes are in green, and Phototransduction genes are in orange. [file 13227_2015_26_MOESM4_ESM.doc]

Cloning and qPCR primers

All primers in 5’-3’ direction

| **Gene homolog** | **Transcriptome identifier*** | **Tree^‡^** |  | **Cloning primers** | **qPCR primers** |
| --- | --- | --- | --- | --- | --- |
| Dac | comp67014 | PIA | F | TGGTGGGTGATTATAGCCATT | GCCATTGGCTAACAGAGGTG |
|  |  |  | R | TCTGCAATGTAGAACAAGTACGG | TGACGCAACAACAACAGGAG |
| Pax6 | DQ139282.1** | PIA | F | ATTCGCATGGGAAATCAGAG | TACTCCATCCCCTCAAAACG |
|  |  |  | R | CACGTCTGGGTAATGGGTTC | GTGCCAACTCCACGATTTTT |
| SO15 | comp15174 | ML | F | CGCATTCCACAGAGGAAACT | CGTTAGGCGCTGTTGGTAAA |
|  |  |  | R | GCTGCCCTGTCTCTTTGTCT | CTTCACCGTCCCAGATTGTC |
| SO17 | comp17226 | ML | F | TTCTCTTGGGGATGGGTATG | CTTCACCGTCCCAGATTGTC |
|  |  |  | R | CGTGTTACCCAGCTTTGGAT | CGTTAGGCGCTGTTGGTAAA |
| Cha | comp959 | ML | F | TTTGACTTTGGCATTGTGGA | GCTGCATAAGACAGGAATCTGG |
|  |  |  | R | TCTTCAGCCGTTGTAGAGCA | GCCGTTGTAGAGCATCTTTGG |
| Da | comp7692 | ML | F | CTGCGCGATCTTTTAGGTTT | GTACCACTGCTGGGTTCCAC |
|  |  |  | R | CCAAGAAGATCAACGCCTTC | TTCCTCCCATGTCATCGTTC |
| EGFR | UN32658 | PIA | F | TGGAGTCAGTGCCGTAATTG | TGGAGTCAGTGCCGTAATTG |
|  |  |  | R | ACGACTCATGACAAGCTGGA | ACGACTCATGACAAGCTGGA |
| Elav | KP965571** | ML | F | AGCGCTGGTGGTATCAAAAT | AGCGCTGGTGGTATCAAAAT |
|  |  |  | R | TCCTATGGCTGGTGATCTGTT | TCCTATGGCTGGTGATCTGTT |
| Sv | comp17488 | ML | F | ACCGGCTGTTAGCTGAGTGT | GCTGCAGACAGAGCAAAGCA |
|  |  |  | R | ATTGGCCATTCAGATGATCC | GGTGTGAAGGCGTGTGTGT |
| Sina | comp44186 | ML | F | CGAATGGAGCCCAGTTCTT | CGAATGGAGCCCAGTTCTT |
|  |  |  | R | CAGCAGAATACAGCATCAGC | CAGCAGAATACAGCATCAGC |
| Calx | comp15223 | ML | F | AGAGGATGGAACAGCCAATG | ACGTGTGGTTGGTACAGATGG |
|  |  |  | R | TGGTCTGCCTAGAAGGGCTA | CCCAAGTTGTAGCCGCATT |
| Ops | UN14183 | PIA | F | CACCCTCACTGGGATCAGTT | GCTCCAAATTCCCAATACCC |
|  |  |  | R | ATAAAACCACCCACCGCATA | GGACACTGCGTTCTCCAAGT |
| PLC | UN08510 | PIA | F | TCGGGCCTTTTACAGAGATG | GTTGCAATTGAACGCCTCTG |
|  |  |  | R | ACTCAAATGGGACGAGGATG | GATCCTGCCGCTTCTATCTGTT |
| PKC | UN14200 | PIA | F | ACATTGGCAGTCTGTGCAAC | CACCACCTTATCCCAACCAG |
|  |  |  | R | CCCTGATTATGTGGAAGCTGA | TCAGGAAGAGGGTCATACGG |

*All transcriptome sequences from .

**Genbank accession number

**^‡^** All PIA trees from , all ML trees can be found in Additional File 3
