# Supplementary material for: Sexually dimorphic gene expression in the lateral eyes of Euphilomedes carcharodonta (Ostracoda, Pancrustacea)
Source: EvoDevo. 2015 Nov 10;6:34. doi: 10.1186/s13227-015-0026-2 (PMC4641368; doi:10.1186/s13227-015-0026-2)
Supplement: Supplementary file 6 — 10.1186/s13227-015-0026-2: Ratio of means for early embryos. The ratio of means for early embryos vs. all other stages for each gene is shown here. Red numbers and pink numbers indicate genes/stages where Tukey’s test showed significantly higher expression in embryos. Black, blue, and purple numbers indicate genes/stages where Tukey’s test showed significantly lower stages in embryos. Specification genes are highlighted in blue, Determination/Patterning genes in green, and Phototransduction genes in orange. [file 13227_2015_26_MOESM6_ESM.pdf]

	Specification				Determination/Patterning						Phototransduction			
	**dac**	**Pax6**	**SO15**	**SO17**	**chaoptic**	**daless**	**egfr**	**elav**	**Shaven**	**Sina**	**Calx**	**opsin**	**PLC**	**PKC**
**L Emb**	0.065585	8.3110944	0.0513496	0.0848377	0.0265815	0.7333002	0.2589478	0.1236839	0.6913015	0.7012687	1.7692432	0.5072183	1.2260321	1.3398695
**F IV**	0.000260496	2.2979467	0.0001316	2.415E-05	0.000414	0.0139626	2.690629	0.6952908	0.1242955	0.3034163	52.762109	0.6495407	0.4950997	0.0094957
**F V**	3.96413E-06	2.6989138	8.353E-05	0.0001112	0.0014627	0.0184228	0.4311676	0.0246868	0.3151341	0.6874172	0.7436616	6.0926357	0.3956618	0.6720039
**F A**	0.000231839	8.9023671	6.847E-05	0.0001133	0.0027391	0.0975516	0.1676129	0.031081	0.0909431	0.1329012	42.362175	4.6474439	25.342169	0.5806832
**M IV**	0.003060086	2.6215491	2.246E-05	2.349E-05	0.0102762	0.0405181	2.3368846	0.0448992	1.8187974	1.2627243	1.6953142	0.9673351	0.1620244	0.0041004
**M V**	0.000280075	1.7921325	0.0002239	0.0001144	0.0487452	0.0238001	1.1615074	0.0651266	5.3077592	1.6325559	13.259613	68.169562	0.6311506	0.0022918
**M A**	0.000529168	48.44188	0.0001361	2.761E-05	0.0035791	0.0566249	0.9233484	0.0523829	0.7728204	0.7503978	393.2331	4091.1258	111.21108	3.3850331

early and late embryos significantly higher

early embryos significantly higher

early and late embryos significantly lower

early embryos significantly lower

late embs significantly lower
