# Supplementary material for: Sexually dimorphic gene expression in the lateral eyes of Euphilomedes carcharodonta (Ostracoda, Pancrustacea)
Source: EvoDevo. 2015 Nov 10;6:34. doi: 10.1186/s13227-015-0026-2 (PMC4641368; doi:10.1186/s13227-015-0026-2)
Supplement: Supplementary file 7 — 10.1186/s13227-015-0026-2: Genes without significant sex/stage differences in eye expression. Average qPCR values for genes without significant differences in expression between the sexes. Ec-Pax-6, Ec-SO17, Ec-Da, Ec-Elav, and Ec-PKC did not show significant differences in pairwise comparisons of expression levels between sexes and stages. Error bars represent standard error. Asterisks highlight genes that showed significance in ANOVA, but not significant pairwise differences using Box-Cox tests. Specification gene homologs are in blue, determination/patterning gene homologs are in green, and phototransduction gene homologs are in orange. Lighter colors denote earlier developmental timepoints. Due to the large variation in expression levels between genes, the Y-axis is log-scaled. [file 13227_2015_26_MOESM7_ESM.pptx]

## Slide 1
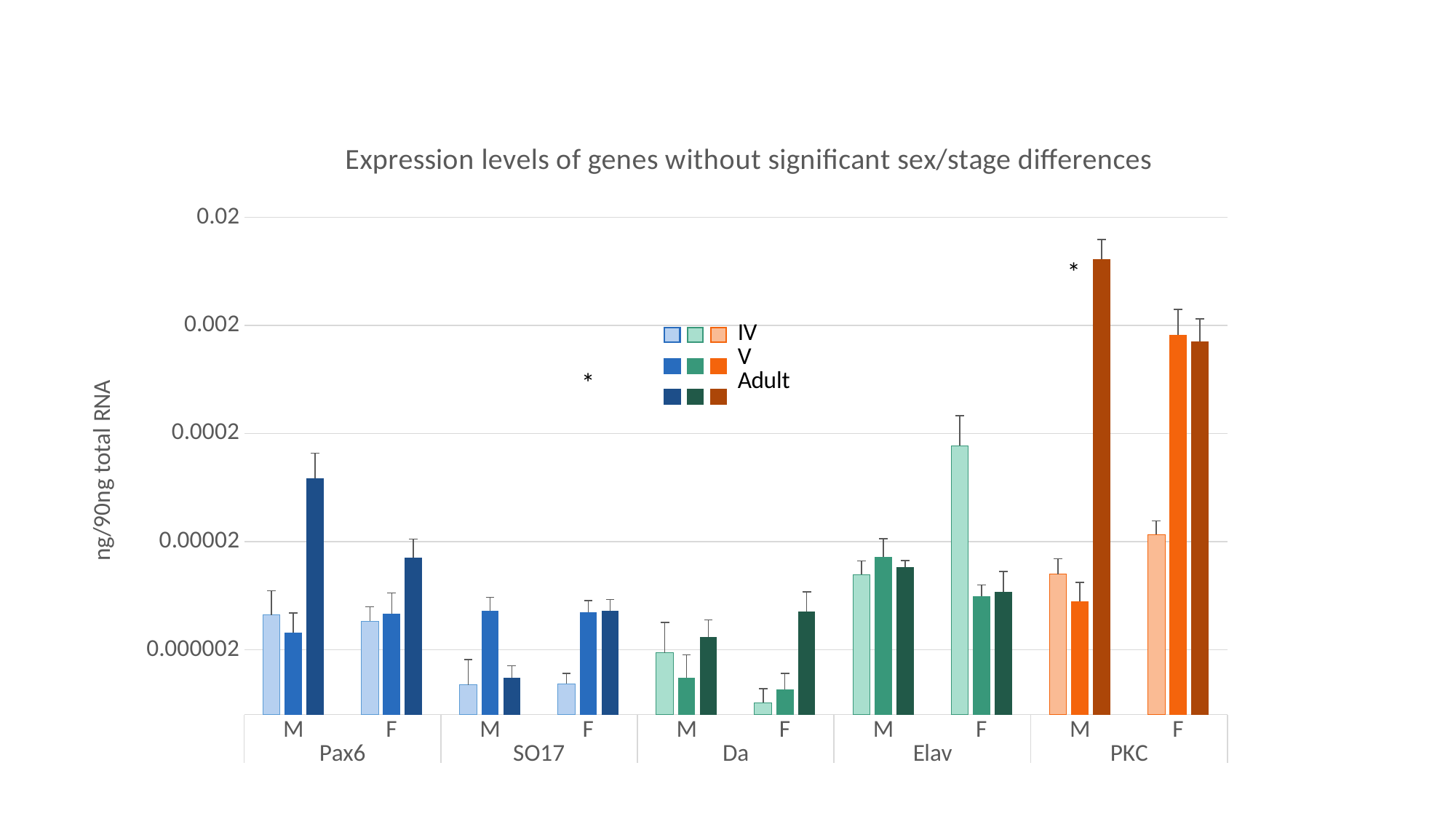

### Chart: Expression levels of genes without significant sex/stage differences
| Category | IV | V | Adult |
|---|---|---|---|
| M | 4.1932356243497894e-06 | 2.8665622613009165e-06 | 7.748403982849927e-05 |
| F | 3.6756252266637984e-06 | 4.316982452810942e-06 | 1.4239566271635742e-05 |
| M | 9.419423219366878e-07 | 4.585265708381944e-06 | 1.1070235957415448e-06 |
| F | 9.683698199684994e-07 | 4.45990495013476e-06 | 4.542181908088632e-06 |
| M | 1.8722199050512287e-06 | 1.0997301331173898e-06 | 2.6164687805797056e-06 |
| F | 6.451703864512949e-07 | 8.512619620447816e-07 | 4.5075705651299405e-06 |
| M | 9.924175067139554e-06 | 1.4395075554473995e-05 | 1.1578323692339517e-05 |
| F | 0.00015368175386037153 | 6.234302710774147e-06 | 6.869911316525545e-06 |
| M | 1.000002297774395e-05 | 5.5891217665504765e-06 | 0.008255406661583756 |
| F | 2.3157981511675675e-05 | 0.0016388808105825667 | 0.0014161681255133141 |*
*
