# Supplementary material for: Sexually dimorphic gene expression in the lateral eyes of Euphilomedes carcharodonta (Ostracoda, Pancrustacea)
Source: EvoDevo. 2015 Nov 10;6:34. doi: 10.1186/s13227-015-0026-2 (PMC4641368; doi:10.1186/s13227-015-0026-2)
Supplement: Supplementary file 8 — 10.1186/s13227-015-0026-2: ANOVA results. ANOVAs were run for the expression values for each gene and reported here. Red numbers indicate ANOVA p-values < 0.05. Asterisks indicate significant pairwise differences between at least one sex/stage by Box-Cox analysis. Specification genes are highlighted in blue, Determination/Patterning genes in green, and Phototransduction genes in orange. [file 13227_2015_26_MOESM8_ESM.pptx]

## Slide 1
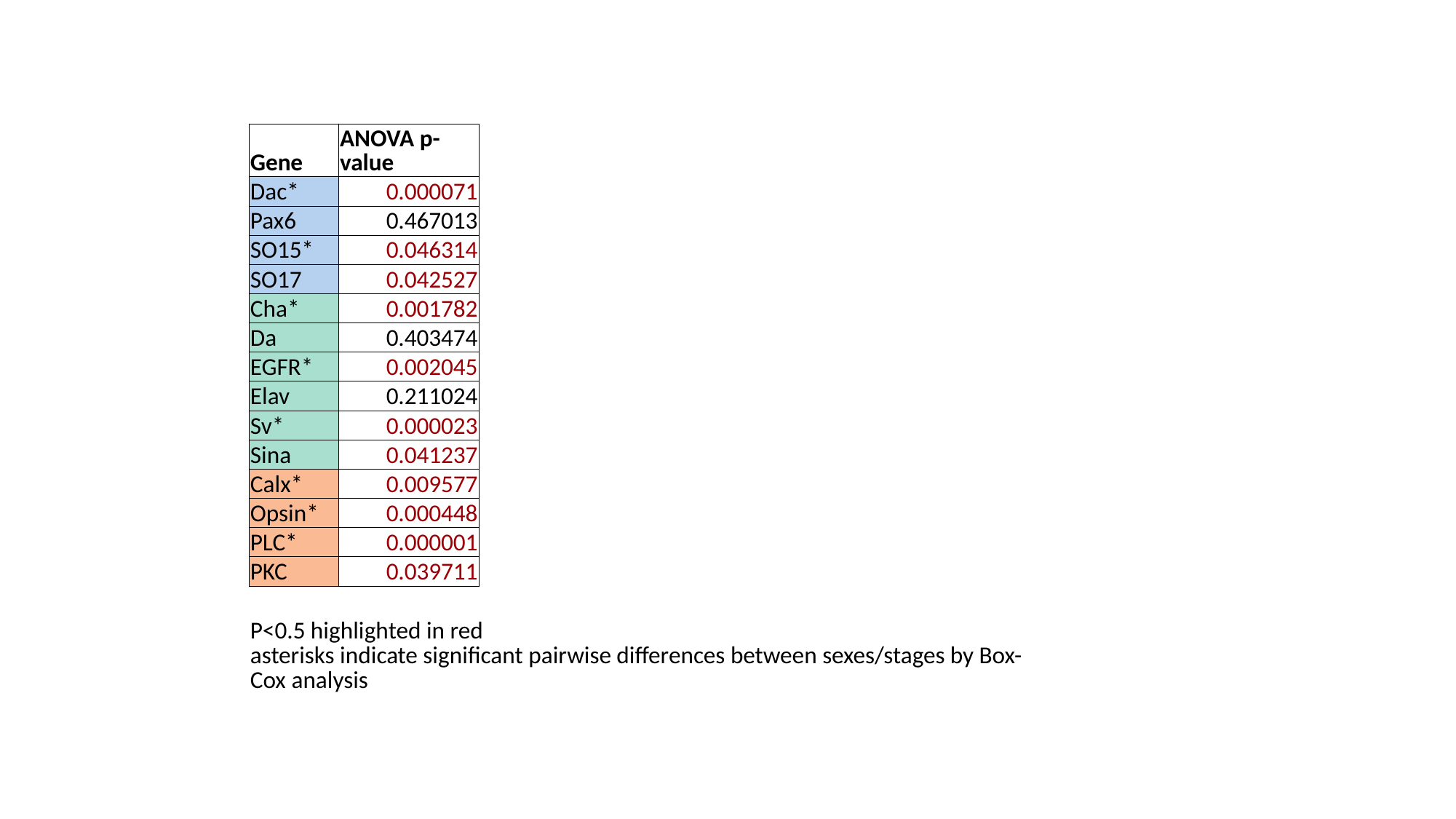

| Gene | ANOVA p-value | | | | | | | |
| --- | --- | --- | --- | --- | --- | --- | --- | --- |
| Dac\* | 0.000071 | | | | | | | |
| Pax6 | 0.467013 | | | | | | | |
| SO15\* | 0.046314 | | | | | | | |
| SO17 | 0.042527 | | | | | | | |
| Cha\* | 0.001782 | | | | | | | |
| Da | 0.403474 | | | | | | | |
| EGFR\* | 0.002045 | | | | | | | |
| Elav | 0.211024 | | | | | | | |
| Sv\* | 0.000023 | | | | | | | |
| Sina | 0.041237 | | | | | | | |
| Calx\* | 0.009577 | | | | | | | |
| Opsin\* | 0.000448 | | | | | | | |
| PLC\* | 0.000001 | | | | | | | |
| PKC | 0.039711 | | | | | | | |
| | | | | | | | | |
| P<0.5 highlighted in red | | | | | | | | |
| asterisks indicate significant pairwise differences between sexes/stages by Box-Cox analysis | | | | | | | | |
| | | | | | | | | |
