# Supplementary material for: Sexually dimorphic gene expression in the lateral eyes of Euphilomedes carcharodonta (Ostracoda, Pancrustacea)
Source: EvoDevo. 2015 Nov 10;6:34. doi: 10.1186/s13227-015-0026-2 (PMC4641368; doi:10.1186/s13227-015-0026-2)
Supplement: Supplementary file 9 — 10.1186/s13227-015-0026-2: Ratio of means for juvenile and adult stages. We report the results of ratio of means analysis here. Comparisons that were significant in pairwise Tukey’s t-tests (adjusted for multiple comparisons) are blue (p < 0.5), green (p < 0.01) or red (p < 0.001). Specification genes are highlighted in blue, Determination/Patterning genes in green, and Phototransduction genes in orange. [file 13227_2015_26_MOESM9_ESM.pdf]

Specification

Dac	F IV	F V	F Adult	M IV	M V	M Adult
F IV	1.00	65.65	1.12	0.09	0.93	0.49
F V	0.02	1.00	0.02	0.00	0.01	0.01
F Adult	0.89	58.39	1.00	0.08	0.83	0.44
M IV	11.74	771.10	13.21	1.00	10.93	5.78
M V	1.07	70.56	1.21	0.09	1.00	0.53
M Adult	2.03	133.35	2.28	0.17	1.89	1.00

Pax6	F IV	F V	F Adult	M IV	M V	M Adult
F IV	1.00	0.85	0.26	0.88	1.28	0.05
F V	1.17	1.00	0.30	1.03	1.50	0.06
F Adult	3.88	3.31	1.00	3.40	4.97	0.18
M IV	1.14	0.97	0.29	1.00	1.46	0.05
M V	0.78	0.67	0.20	0.68	1.00	0.04
M Adult	21.08	17.99	5.44	18.48	27.05	1.00

SO15	F IV	F V	F Adult	M IV	M V	M Adult
F IV	1.00	1.58	1.92	5.86	0.59	0.97
F V	0.63	1.00	1.22	3.71	0.37	0.61
F Adult	0.52	0.82	1.00	3.05	0.31	0.50
M IV	0.17	0.27	0.33	1.00	0.10	0.17
M V	1.70	2.69	3.27	9.96	1.00	1.65
M Adult	1.03	1.63	1.99	6.06	0.61	1.00

SO17	F IV	F V	F Adult	M IV	M V	M Adult
F IV	1.00	0.22	0.21	1.03	0.21	0.88
F V	4.60	1.00	0.98	4.73	0.97	4.03
F Adult	4.69	1.02	1.00	4.82	0.99	4.10
M IV	0.97	0.21	0.21	1.00	0.21	0.85
M V	4.73	1.03	1.01	4.86	1.00	4.14
M Adult	1.14	0.25	0.24	1.17	0.24	1.00

Determination/Patterning

Chaoptic	F IV	F V	F Adult	M IV	M V	M Adult
F IV	1.00	0.28	0.15	0.04	0.01	0.12
F V	3.54	1.00	0.53	0.14	0.03	0.41
F Adult	6.62	1.87	1.00	0.27	0.06	0.77
M IV	24.83	7.02	3.75	1.00	0.21	2.87
M V	117.82	33.32	17.79	4.74	1.00	13.61
M Adult	8.66	2.45	1.31	0.35	0.07	1.00

Daless	F IV	F V	F Adult	M IV	M V	M Adult
F IV	1.00	0.76	0.14	0.34	0.59	0.25
F V	1.32	1.00	0.19	0.45	0.77	0.33
F Adult	6.98	5.28	1.00	2.40	4.09	1.72
M IV	2.90	2.20	0.42	1.00	1.70	0.72
M V	1.70	1.29	0.24	0.59	1.00	0.42
M Adult	4.06	3.07	0.58	1.40	2.38	1.00

EGFR	F IV	F V	F Adult	M IV	M V	M Adult
F IV	1.00	6.24	16.05	1.15	2.32	2.91
F V	0.16	1.00	2.57	0.18	0.37	0.47
F Adult	0.06	0.39	1.00	0.07	0.14	0.18
M IV	0.87	5.42	13.94	1.00	2.01	2.53
M V	0.43	2.69	6.93	0.50	1.00	1.26
M Adult	0.34	2.14	5.51	0.40	0.80	1.00

Elav	F IV	F V	F Adult	M IV	M V	M Adult
F IV	1.00	28.17	22.39	15.50	10.68	13.27
F V	0.04	1.00	0.80	0.55	0.38	0.47
F Adult	0.04	1.26	1.00	0.69	0.48	0.59
M IV	0.06	1.82	1.44	1.00	0.69	0.86
M V	0.09	2.64	2.10	1.45	1.00	1.24
M Adult	0.08	2.12	1.69	1.17	0.80	1.00

Shaven	F IV	F V	F Adult	M IV	M V	M Adult
F IV	1.00	0.39	1.37	0.07	0.02	0.16
F V	2.54	1.00	3.47	0.17	0.06	0.41
F Adult	0.73	0.29	1.00	0.05	0.02	0.12
M IV	14.62	5.76	20.01	1.00	0.34	2.35
M V	42.70	16.82	58.42	2.92	1.00	6.87
M Adult	6.22	2.45	8.51	0.43	0.15	1.00

Sina	F IV	F V	F Adult	M IV	M V	M Adult
F IV	1.00	0.44	2.28	0.24	0.19	0.40
F V	2.26	1.00	5.17	0.54	0.42	0.91
F Adult	0.44	0.19	1.00	0.11	0.08	0.18
M IV	4.16	1.84	9.49	1.00	0.77	1.68
M V	5.38	2.38	12.27	1.29	1.00	2.17
M Adult	2.48	1.09	5.65	0.59	0.46	1.00

Phototransduction

Calx	F IV	F V	F Adult	M IV	M V	M Adult
F IV	1.00	70.96	1.25	31.13	3.98	0.13
F V	0.01	1.00	0.02	0.44	0.06	0.00
F Adult	0.80	56.97	1.00	25.00	3.19	0.11
M IV	0.03	2.28	0.04	1.00	0.13	0.00
M V	0.25	17.83	0.31	7.82	1.00	0.03
M Adult	7.45	528.85	9.28	232.04	29.66	1.00

Opsin	F IV	F V	F Adult	M IV	M V	M Adult
F IV	1.00	0.11	0.14	0.67	0.01	0.00
F V	9.38	1.00	1.31	6.30	0.09	0.00
F Adult	7.15	0.76	1.00	4.80	0.07	0.00
M IV	1.49	0.16	0.21	1.00	0.01	0.00
M V	104.94	11.19	14.67	70.47	1.00	0.02
M Adult	6297.58	671.39	880.47	4229.00	60.01	1.00

PKC	F IV	F V	F Adult	M IV	M V	M Adult
F IV	1.00	0.01	0.02	2.32	4.15	0.00
F V	70.76	1.00	1.16	164.06	293.68	0.20
F Adult	61.14	0.86	1.00	141.77	253.77	0.17
M IV	0.43	0.01	0.01	1.00	1.79	0.00
M V	0.24	0.00	0.00	0.56	1.00	0.00
M Adult	356.42	5.04	5.83	826.43	1479.33	1.00

PLC	F IV	F V	F Adult	M IV	M V	M Adult
F IV	1.00	1.25	0.02	3.05	0.78	0.00
F V	0.80	1.00	0.02	2.44	0.63	0.00
F Adult	51.26	64.07	1.00	156.34	40.16	0.23
M IV	0.33	0.41	0.01	1.00	0.26	0.00
M V	1.28	1.60	0.02	3.89	1.00	0.01
M Adult	224.94	281.18	4.39	686.07	176.23	1.00

Color	P <
Blue	0.05
Green	0.01
Red	0.001
